# Somatic symptoms mediate the association between subclinical anxiety and depressive symptoms and its neuroimaging mechanisms

**DOI:** 10.1186/s12888-022-04488-9

**Published:** 2022-12-29

**Authors:** Zhifei Kong, Ximei Zhu, Suhua Chang, Yanping Bao, Yundong Ma, Wenwen Yu, Ran Zhu, Qiqing Sun, Wei Sun, Jiahui Deng, Hongqiang Sun

**Affiliations:** 1 Peking University Sixth Hospital, Peking University Institute of Mental Health, NHC Key Laboratory of Mental Health (Peking University), National Clinical Research Center for Mental Disorders (Peking University Sixth Hospital), Beijing, 100191 China; 2 National Institute on Drug Dependence and Beijing Key Laboratory of Drug Dependence, Peking University, Beijing, 100191 China; 3 School of Public Health, Peking University, Beijing, 100191 China

**Keywords:** Subclinical anxiety symptoms, Subclinical depressive symptoms, Somatic symptoms, Resting-state functional connectivity, Mediation analysis

## Abstract

**Background:**

Subclinical anxiety, depressive and somatic symptoms appear closely related. However, it remains unclear whether somatic symptoms mediate the association between subclinical anxiety and depressive symptoms and what the underlying neuroimaging mechanisms are for the mediating effect.

**Methods:**

Data of healthy participants (*n* = 466) and participants in remission of major depressive disorder (*n* = 53) were obtained from the Human Connectome Project. The Achenbach Adult Self-Report was adopted to assess anxiety, depressive and somatic symptoms. All participants completed four runs of resting-state functional magnetic resonance imaging. Mediation analyses were utilized to explore the interactions among these symptoms and their neuroimaging mechanisms.

**Results:**

Somatic symptoms partially mediated the association between subclinical anxiety and depressive symptoms in healthy participants (anxiety→somatic→depression: effect: 0.2785, Boot 95% CI: 0.0958–0.3729; depression→somatic→anxiety: effect: 0.0753, Boot 95% CI: 0.0232–0.1314) and participants in remission of MDD (anxiety→somatic→depression: effect: 0.2948, Boot 95% CI: 0.0357–0.7382; depression→somatic→anxiety: effect: 0.0984, Boot 95% CI: 0.0007–0.2438). Resting-state functional connectivity (FC) between the right medial superior frontal gyrus and the left thalamus and somatic symptoms as chain mediators partially mediated the effect of subclinical depressive symptoms on subclinical anxiety symptoms in healthy participants (effect: 0.0020, Boot 95% CI: 0.0003–0.0043). The mean strength of common FCs of subclinical depressive and somatic symptoms, somatic symptoms, and the mean strength of common FCs of subclinical anxiety and somatic symptoms as chain mediators partially mediated the effect of subclinical depressive symptoms on subclinical anxiety symptoms in remission of MDD (effect: 0.0437, Boot 95% CI: 0.0024–0.1190). These common FCs mainly involved the insula, precentral gyri, postcentral gyri and cingulate gyri. Furthermore, FC between the triangular part of the left inferior frontal gyrus and the left postcentral gyrus was positively associated with subclinical anxiety, depressive and somatic symptoms in remission of MDD (FDR-corrected *p* < 0.01).

**Conclusions:**

Somatic symptoms partially mediate the interaction between subclinical anxiety and depressive symptoms. FCs involving the right medial superior frontal gyrus, left thalamus, triangular part of left inferior frontal gyrus, bilateral insula, precentral gyri, postcentral gyri and cingulate gyri maybe underlie the mediating effect of somatic symptoms.

**Supplementary Information:**

The online version contains supplementary material available at 10.1186/s12888-022-04488-9.

## Background

Subclinical anxiety/depression occurs when individuals experience anxiety/depressive symptoms that do not meet the diagnostic criteria for anxiety disorders (ANX)/major depressive disorder (MDD) [1–6]. Subclinical anxiety and depression increased the risk of ANX and MDD [3, 6–9], which displayed high prevalence [10–12]. Notably, 23–39% of adolescents and young adults suffered from subclinical anxiety or depression [13–15], which contributed to the increased risk of suicide and functional impairment [15, 16]. Subclinical anxiety and depression often coexist with a prevalence of their comorbidity ranging from 4.60–12.98%, leading to more severe symptoms, work impairment and worse treatment outcomes [1, 15, 17, 18], which needs to be noted.

The global age-standardized prevalence of depressive disorder (MDD and dysthymia) was 3.44%, which caused disability-adjusted life years accounting for 37.3% of that due to mental disorders, ranking first among all mental disorders [12]. 85% of patients recovering from MDD recurred within 15 years [19], and anxiety and depressive symptoms in remission of depression increased the risk of its relapse [20, 21]. Thus, it is necessary to get a better understanding of these symptoms in remission of MDD, which may contribute to making effective interventions to relieve them and prevent MDD relapse.

Somatic symptoms are closely related to anxiety and depressive symptoms. Patients with ANX or MDD often experienced somatic symptoms [22, 23], which caused patients to seek medical visits, increased suicide rates, reduced antidepressant efficacy, and worsened outcomes [24–27]. Additionally, somatic symptoms were moderately correlated with subclinical anxiety and depressive symptoms with a correlation coefficient greater than 0.4 [17]. Neuroimaging studies found that the brain regions associated with these symptoms overlapped [17, 18, 28, 29]. The volume of the amygdala was negatively correlated with the severity of subclinical anxiety [18], and this region was also part of the neural circuit framework for somatosensory amplification [28]. The gray matter of the postcentral gyrus was positively correlated with subclinical depression [17], and the regional homogeneity and amplitude of low-frequency fluctuation in this region were negatively related to somatic symptoms [29]. However, the role of somatic symptoms in the association between subclinical anxiety and depressive symptoms remains unclear.

Based on the above, we aimed to assess the mediating effect of somatic symptoms on the association between subclinical anxiety and depressive symptoms with simple mediation models and to explore resting-state functional connectivities (FCs) underlying the mediating effect with chain mediation models [30, 31]. Identifying alterations in the brain before the onset of the disorders may contribute to early detection and intervention [32].

## Methods

### Participants

Our data were obtained from the Human Connectome Project (HCP)-Young Adult database [33]. HCP data were provided by researchers at the University of Southern California, Martinos Center for Biomedical Imaging at Massachusetts General Hospital, Washington University, and the University of Minnesota. We included data of two kinds of people: healthy participants (HP) and participants in remission of MDD (MDP), and the flow charts of identifying the participants in our study are shown in Additional file 1: Fig. S1 and Fig. S2.

Previous studies defined a scale item score ≥ 1 as one symptom [34–36] and defined at least one anxiety symptom/depressive symptom which did not meet the diagnostic criteria for ANX/MDD as subclinical anxiety/depression [1–6]. The DSM-IV Depressive Problems subscale and DSM-IV Anxiety Problems subscale used in the HCP were DSM-oriented scales consisting of items that were quite consistent with the DSM-IV categories. Furthermore, subjects in the HCP did not meet the diagnostic criteria for ANX/MDD at the time of recruitment. Therefore, we considered anxiety score ≥ 1 and depression score ≥ 1 as subclinical anxiety and depressive symptoms.

Beyond the inclusion and exclusion criteria of the HCP [37], participants with anxiety score ≥ 1 and depression score ≥ 1 were included in our study if they did not meet any of the following criteria: positive breathalyzer test results, positive drug test results, alcohol abuse, alcohol dependence or marijuana dependence. Additionally, participants with histories of panic disorder/agoraphobia were excluded in the HP. All participants completed four runs of resting-state functional magnetic resonance imaging (rs-fMRI) at Washington University. They have signed full written informed consents, and all study procedures were executed following the ethical standards of Washington University Institutional Review Board [37].

### Measurements

#### Assessment for psychiatric history

All participants underwent a comprehensive assessment of their psychiatric and substance use history over the telephone using the Semi-Structured Assessment for the Genetics of Alcoholism (SSAGA), which assessed a range of diagnostic categories (substance, mood, anxiety, eating disorders, adult attention-deficit/hyperactivity disorder and antisocial personality disorder) using both the Diagnostic and Statistical Manual of Mental Disorders, Fourth Edition (DSM-IV) and either the Research Diagnostic Criteria or International Classification of Diseases and provided information about both current and lifetime experiences. The SSAGA is a well-validated diagnostic instrument and has been used in numerous previous large-scale studies [38, 39].

#### Assessment for anxiety, depressive and somatic symptoms

The anxiety, depressive and somatic symptoms were assessed by the DSM-IV Anxiety Problems subscale, DSM-IV Depressive Problems subscale and Somatic Complaints subscale, based on the Achenbach Adult Self-Report (ASR) for ages 18–59, and all the items in these subscales are listed in Table S1 of Additional file 1 [40]. The score of each item ranges from 0 to 2, and “0” means “Not True”, and “1” means “Somewhat or Sometimes True”, and “2” means “Very True or Often True” over the past six months. The DSM-IV Anxiety Problems subscale and DSM-IV Depressive Problems subscale are components of the DSM-oriented scales, and the Somatic Complaints subscale is a component of the syndrome scales. The ASR profile is characterized as a DSM-oriented scale consisting of items that are considered to be quite consistent with the DSM-IV categories by experts from many cultures. All ASR subscales exhibit excellent test-retest reliability and internal consistency [40]. Both DSM-oriented scales and syndrome scales are equally predictive of affective disorders [41–43].

### Magnetic resonance imaging data

#### Magnetic resonance imaging data preprocessing

The rs-fMRI data were acquired with a gradient-echo echo-planar imaging sequence on a customized Siemens Skyra 3 T scanner with a 32-channel head coil (repetition time = 720 ms, echo time = 33.1 ms, echo spacing = 0.58 ms, field of view = 208 × 180 mm, matrix = 104 × 90, 72 slices, slice thickness = 2.0 mm, voxel size = 2.0 × 2.0 × 2.0 mm, flip angle = 52°). Each run of rs-fMRI lasted approximately 15 minutes, and each session consisted of a run with a phase encoding in a right-to-left direction and another run with a phase encoding in a left-to-right direction. Two sessions were completed during a two-day visit. The MRI data were preprocessed by the HCP consortium with its unified methods with the FMRIB Software Library (FSL), FreeSurfer, and Connectome Workbench software [44].

#### Construction of the whole-brain functional network

The gray matter of the whole brain was parcellated into 250 functional regions of interest based on the Shen atlas following preprocessing of the raw imaging data, and their anatomical names were found in Anatomical Automatic Labeling-2 atlas [30, 45, 46]. The Shen atlas is a validated method to parcellate brain regions [47, 48]. The total number of FCs between 250 regions is 31,125 (250 × 249/2 = 31,125). The average signal value of all voxels in each region of interest was calculated to extract the time series of this region. For each participant, correlations between the blood oxygen level-dependent signals of every pair of regions were computed via Pearson correlation analysis, and their normality was improved by z-transformation.

### Statistical analysis

#### Exploring the mediating effect of somatic symptoms on the association between subclinical anxiety and depressive symptoms

Spearman partial correlation analysis was used to analyze the correlations among subclinical anxiety, depressive and somatic symptoms after controlling for covariates including age, gender, race, handedness [49], education, parental history of neuropsychiatric disorders, times smoked, times used illicit drug and times used marijuana in the HP. In addition, a history of panic disorder/agoraphobia was added to the analysis as a covariate in the MDP. Then, a simple mediation model (PROCESS Model 4) [50] was used to investigate the mediating effect of somatic symptoms on the association between subclinical anxiety and depressive symptoms. In the model, somatic symptoms were the mediator, subclinical anxiety and depressive symptoms were the independent/dependent variables and the aforementioned covariates were the control variables. The percentile bootstrap analysis with 5000 bootstrap samples was used to estimate the mediating effect, and the result with bootstrap 95% confidence interval (Boot 95% CI) that did not contain zero was regarded as significant.

#### Identifying FCs associated with subclinical anxiety, depressive or somatic symptoms

To identify FCs associated with subclinical anxiety symptoms, a general linear regression model was established using the network-based statistic toolkit. In this model, subclinical anxiety symptoms were the dependent variable, FC strength was the independent variable, and the aforementioned control variables were covariates. To address the issue of multiple comparisons, *p* values corrected by the False Discovery Rate (FDR) less than 0.05 were considered significant [51]. The same method was employed to identify FCs associated with subclinical depressive and somatic symptoms.

#### Exploring FCs underlying the mediating effect of somatic symptoms on the association between subclinical anxiety and depressive symptoms

A chain mediation model was established by writing a program in PROCESS [50] to explore FCs underlying the mediating effect of somatic symptoms on the association between subclinical anxiety and depressive symptoms. In this model, the mean strength of the common FCs of subclinical anxiety and somatic symptoms (AS-FC), somatic symptoms and the mean strength of the common FCs of subclinical depressive and somatic symptoms (DS-FC) were the chain mediators [30, 31], subclinical anxiety and depressive symptoms were the independent/dependent variables and the same covariates mentioned above were control variables. The mediating effect value with a Boot 95% CI that did not contain zero was regarded as significant.

## Results

### Demographics

A total of 466 subjects (female: 61.4%; age: 22–36 years) and 53 subjects (female: 67.9%; age: 22–36 years) were included in the HP and MDP, respectively. Demographic data, including age, gender, race, handedness, education, parental history of neuropsychiatric disorders, times smoked, times used illicit drug and times used marijuana and history of panic disorder/agoraphobia, are shown in Table 1.

**Table 1:** Demographic characteristics

Characteristics	N (%)	
	HP (*n* = 466)	MDP (*n* = 53)
Age, mean ± SD, years	28.81 ± 3.778	28.62 ± 3.914
**Gender:**		
Male	180 (38.6)	17 (32.1)
Female	286 (61.4)	36 (67.9)
**Race:**		
White	363 (77.9)	38 (71.7)
Other	103 (22.1)	15 (28.3)
**Handedness:**		
Right handedness	294 (63.1)	34 (64.2)
Non-right handedness	172 (36.9)	19 (35.8)
**Education, years:**		
≤11	7 (1.5)	1 (1.9)
12	58 (12.4)	5 (9.4)
13	24 (5.2)	3 (5.7)
14	52 (11.2)	9 (17.0)
15	31 (6.7)	3 (5.7)
16	221 (47.4)	25 (47.2)
≥17	73 (15.7)	7 (13.2)
**Parental history of neuropsychiatric disorders:**		
Yes	119 (25.5)	20 (37.7)
No	347 (74.5)	33 (62.3)
**Smoking history:**		
Never smoked	316 (67.8)	30 (56.6)
Experimented 1–19 times	27 (5.8)	6 (11.3)
Experimented 20–99 times	59 (12.7)	3 (5.7)
Regular smoker	64 (13.7)	14 (26.4)
**Illicit drug use:**		
Never used	428 (91.8)	42 (79.2)
1–2 times	23 (4.9)	6 (11.3)
3–10 times	10 (2.1)	5 (9.4)
11–25 times	3 (0.6)	–
26–100 times	2 (0.4)	–
> 100 times (only male)	–	–
**Marijuana use:**		
Never used	297 (63.7)	27 (50.9)
1–5 times	100 (21.5)	15 (28.3)
6–10 times	26 (5.6)	7 (13.2)
11–100 times	29 (6.2)	3 (5.7)
101–999 times	10 (2.1)	1 (1.9)
≥1000 times	4 (0.9)	–
**History of panic disorder/agoraphobia:**		
Yes	–	19 (35.8)
No	–	34 (64.2)
DSM-IV anxiety problems raw score, median (interquartile range)	3.5 (3)	6 (3)
DSM-IV depressive problems raw score, median (interquartile range)	3 (4)	6 (6)
Adult self-report somatic complaints raw score, median (interquartile range)	3 (3)	3 (5)

Note: ***HP*** **healthy participants;** ***MDP*** **participants in remission of major depressive disorder**

### Associations among subclinical anxiety, depressive and somatic symptoms

**Fig. 1:**
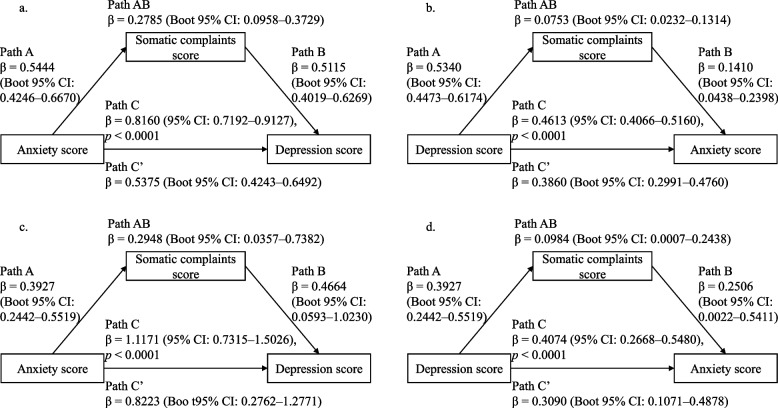
The mediating effects of somatic symptoms on the association between subclinical anxiety and depressive symptoms. Legend: a and b: the mediating effect of somatic symptoms on the association between subclinical anxiety and depressive symptoms in healthy participants; c and d: the mediating effect of somatic symptoms on the association between subclinical anxiety and depressive symptoms in remission of major depressive disorder; Path A: the effect of the independent variable on the mediator; Path B: the effect of the mediator on the dependent variable; Path AB: the indirect path in this model; Path C′: the direct effect of the independent variable on the dependent variable after controlling for the effect of the mediator; Path C: the total effect of the independent variable on the dependent variable; β shows the regression coefficient of the effect of one variable on the other; Boot 95% CI: Bootstrap 95% confidence interval

Subclinical anxiety, depressive and somatic symptoms were significantly interrelated with each other (*p* < 0.001; Additional file 1: Table S2). In both the HP and MDP, somatic symptoms significantly mediated the association between subclinical anxiety and depressive symptoms in both directions (Fig. 1).

### FCs associated with subclinical anxiety, depressive and somatic symptoms

**Fig. 2:**
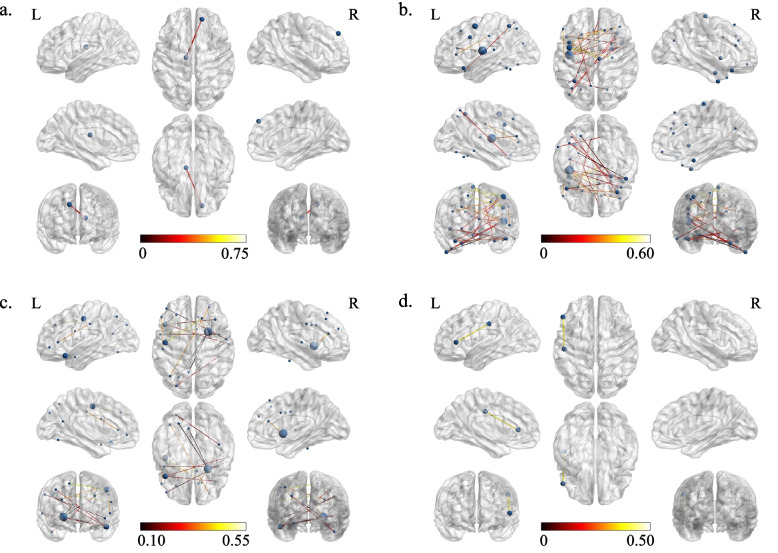
Common functional connectivities (FCs) of subclinical anxiety, depressive and somatic symptoms. Legend: a: the common FC of subclinical depressive and somatic symptoms in healthy participants; b: the common FCs of subclinical anxiety and somatic symptoms in remission of major depressive disorder (MDD); c: the common FCs of subclinical depressive and somatic symptoms in remission of MDD; d: the common FC of subclinical anxiety, depressive and somatic symptoms in remission of MDD; the bar means FC strength (Z-value)

In the HP, FCs associated with subclinical anxiety, depressive and somatic symptoms are shown in Tables S3-S5 of Additional file 1, respectively. An FC between the right medial superior frontal gyrus and the left thalamus was significantly associated with both subclinical depressive and somatic symptoms (Fig. 2a).

In the MDP, FCs associated with subclinical anxiety, depressive and somatic symptoms are shown in Tables S6-S8 in Additional file 1, respectively. We found 24 common FCs associated with both subclinical anxiety and somatic symptoms (Fig. 2b and Additional file 1: Table S9) and 13 common FCs associated with both subclinical depressive and somatic symptoms (Fig. 2c and Additional file 1: Table S10). An FC between the triangular part of the left inferior frontal gyrus and the left postcentral gyrus was significantly positively associated with all three phenotypes (Fig. 2d).

Notably, FCs strength were negatively associated with the three kinds of symptoms in the HP but positively associated with them in the MDP. An FC between the right postcentral gyrus and the left precentral gyrus was associated with somatic symptoms in both the HP and MDP. Nevertheless, no common FC was associated with subclinical anxiety or depressive symptoms in the HP and MDP.

### FCs underlying the mediating effect of somatic symptoms on the association between subclinical anxiety and depressive symptoms

**Fig. 3:**
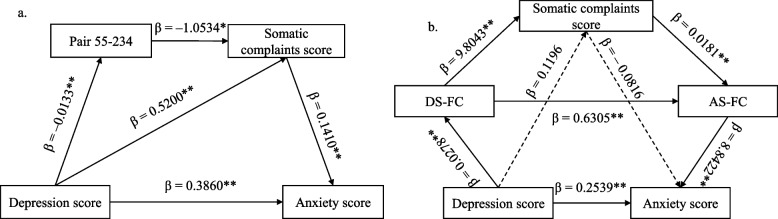
The chain mediation models of the effect of subclinical depressive symptoms on subclinical anxiety symptoms Legend: a: the chain mediation model of the effect of subclinical depressive symptoms on subclinical anxiety symptoms in healthy participants; b: the chain mediation model of the effect of subclinical depressive symptoms on subclinical anxiety symptoms in remission of major depressive disorder; Pair 55–234: the strength of the functional connectivity between the right medial superior frontal gyrus and the left thalamus; DS-FC: the mean strength of common functional connectivities of subclinical depressive and somatic symptoms after removing the functional connectivity between the triangular part of the left inferior frontal gyrus and the left postcentral gyrus; AS-FC: the mean strength of common functional connectivities of subclinical anxiety and somatic symptoms after removing the functional connectivity between the triangular part of the left inferior frontal gyrus and the left postcentral gyrus; β: regression coefficient; *p < 0.05; **p < 0.01.

In the HP, an FC strength between the right medial superior frontal gyrus and the left thalamus and somatic complaints scores were taken as mediators into the chain mediation model (Fig. 3a and Additional file 1: Fig. S3a). The chain indirect path significantly mediated the effect of subclinical depressive symptoms on subclinical anxiety symptoms (mediating effect value: 0.0020, Boot 95% CI: 0.0003–0.0043), but the opposite direction did not yield a significant result (Additional file 1: Table S11 and Table S12).

In the MDP, after removing the FC strength between the triangular part of the left inferior frontal gyrus and the left postcentral gyrus, which associated with the three dimensions of symptoms, we took the mean strength of the remaining 23 common FCs associated with both subclinical anxiety and somatic symptoms (AS-FC), the somatic complaints scores, and the mean strength of the remaining 12 common FCs associated with both subclinical depressive and somatic symptoms (DS-FC) as mediators into the chain mediation model (Fig. 3b and Additional file 1: Fig. S3b). The chain indirect path— depressive symptoms → DS-FC → somatic symptoms → AS-FC → anxiety symptoms— significantly mediated the effect of subclinical depressive symptoms on subclinical anxiety symptoms after controlling for other indirect paths (mediating effect value: 0.0437, Boot 95% CI: 0.0024–0.1190; Additional file 1: Table S13), but the chain indirect path— anxiety symptoms → AS-FC → somatic symptoms → DS-FC → depressive symptoms— was not significant (Additional file 1: Table S14). Moreover, indirect paths that included only AS-FC and DS-FC as mediators significantly mediated the association between subclinical anxiety and depressive symptoms bidirectionally after controlling for the other indirect paths (Additional file 1: Table S13 and Table S14).

## Discussion

In the present study, we found that somatic symptoms partially mediated the association between subclinical anxiety and depressive symptoms bidirectionally in the healthy participants and participants in remission of MDD, which implies that subclinical anxiety and depressive symptoms interact to some extent by affecting somatic symptoms. Furthermore, in the healthy participants, the FC between the right medial superior frontal gyrus and the left thalamus and somatic symptoms as chain mediators partially mediated the effect of subclinical depressive symptoms on subclinical anxiety symptoms. In remission of MDD, the mean strength of common FCs of subclinical depressive and somatic symptoms, somatic symptoms, and the mean strength of common FCs of subclinical anxiety and somatic symptoms as chain mediators partially mediated the effect of subclinical depressive symptoms on subclinical anxiety symptoms; the mean strength of common FCs of subclinical anxiety and somatic symptoms and that of subclinical depressive and somatic symptoms as chain mediators partially mediated the interactions between subclinical anxiety and depressive symptoms. These common FCs mainly involved the insula, precentral gyri, postcentral gyri and cingulate gyri. In addition, the FC between the triangular part of the left inferior frontal gyrus and the left postcentral gyrus was significantly associated with subclinical anxiety, depressive and somatic symptoms in remission of MDD. These FCs mentioned above may explain the relationships among subclinical anxiety, depressive and somatic symptoms. Our findings may improve the understanding of these symptoms.

Subclinical anxiety, depressive and somatic symptoms were found to be correlated with each other in our study, as well as in previous studies [17, 18], which was the premise of the mediating effect of somatic symptoms on the association between subclinical anxiety and depressive symptoms. Actually, we found that somatic symptoms played a mediating role in the association between subclinical anxiety and depressive symptoms, indicating that subclinical anxiety and depressive symptoms interact through somatic symptoms. Several studies also found that somatic symptoms played a mediating effect. Somatic symptoms mediated the relationship between anxiety/depressive symptoms and abdominal pain and the relationship between health anxiety and health-related quality of life [52, 53]. Furthermore, several brain regions related to the three kinds of symptoms were found to be overlapped, such as the amygdala and postcentral gyrus [17, 18, 28, 29], which may provide rational evidence of the relationship among these symptoms. Therefore, somatic symptoms should be considered when coping with subclinical anxiety and depressive symptoms in practice.

The common FC between the right medial superior frontal gyrus and the left thalamus and somatic symptoms as chain mediators partially mediated the effects of subclinical depressive symptoms on subclinical anxiety symptoms in the healthy participants, which reveals that subclinical depressive symptoms may influence the FC and somatic symptoms in sequence and then affect subclinical anxiety symptoms. The superior frontal gyrus was related to emotional regulation [54] and cognitive control [55], which affected depressive symptoms [56, 57]. Additionally, the fractional amplitude of low-frequency fluctuations and short-range positive FC strength of this brain region increased in somatization disorder [58, 59]. These studies support that the right medial superior frontal gyrus is involved in depressive and somatic symptoms. The thalamus activated during experiencing and regulating emotional distress [60] and engaged in subclinical depressive symptoms [61]. The thalamus was also related to sensory processing [62, 63], which might play a role in somatic symptoms. These studies suggest that the left thalamus works on depressive and somatic symptoms. Furthermore, FC strength between the right superior frontal gyrus and the left thalamus significantly reduced in patients with fatigue, euthymic bipolar disorder or schizophrenia [64–66]. Therefore, we consider that the FC between the right medial superior frontal gyrus and the left thalamus may be implicated in subclinical depressive and somatic symptoms. In the chain mediation model, this FC and somatic symptoms played a chain mediating effect on the association between subclinical depressive and anxiety symptoms, which implies that this FC maybe influences the relationships among these behavioral symptoms in healthy people.

The FC between the triangular part in the left inferior frontal gyrus and the left postcentral gyrus was significantly associated with subclinical anxiety, depressive and somatic symptoms in remission of MDD. The functional activity of the triangular part in the left inferior frontal gyrus increased in MDD [67], and the gray matter in the left inferior prefrontal cortex also increased as somatic symptoms worsened [17]. Additionally, the postcentral gyrus, including somatosensory areas of the brain, influenced the integration and processing of incoming and outgoing signals of the body [68], involved in pain and irritable bowel syndrome [69, 70], and played a vital role in emotional processing [71], based on which we can speculate that this brain region may relate to affective and somatic symptoms. The structure and function of this brain region also altered in ANX, MDD and subclinical depression [17, 29, 72–74]. Furthermore, the controllability of a network, including the left superior frontal gyrus, left inferior frontal gyrus, left postcentral gyrus, left insula and left pars triangularis, was significantly reduced in young adult patients with bipolar disorders compared with healthy controls and unaffected siblings or children at high genetic risk, which suggests that these brain regions might contribute to poor emotional control [75]. In summary, the FC between the triangular part of the left inferior frontal gyrus and the left postcentral gyrus may be a potential intervention target for improving subclinical anxiety, depressive and somatic symptoms to prevent MDD relapse/recurrence.

Notably, the mediating effect of somatic symptoms was significant in the simple mediation model, whereas it was insignificant after controlling for the other indirect paths in the chain mediation model in remission of MDD. Furthermore, we found that the mean strength of the common FCs of subclinical anxiety and somatic symptoms and that of subclinical depressive and somatic symptoms as chain mediators independently mediated the interaction between subclinical anxiety and depressive symptoms, and that the mean strength of the common FCs of subclinical depressive and somatic symptoms, somatic symptoms, and the mean strength of the common FCs of subclinical anxiety and somatic symptoms as chain mediators independently mediated the effect of subclinical depressive symptoms on subclinical anxiety symptoms in remission of MDD. These common FCs mainly involved the insula, precentral gyri, postcentral gyri and cingulate gyri, which were related to anxiety, depressive and somatic symptoms [17, 29, 74, 76–82]. Therefore, the common FCs maybe underlie the mediating effect of somatic symptoms on the association between subclinical anxiety and depressive symptoms and affect the relationships among these symptoms in remission of MDD. However, it should be cautious to this speculation because of the small sample size.

We found a positive correlation between FC strength and the severity of subclinical depressive symptoms in remission of MDD, which was similar to the results of another study with subjects from the HCP [30]. However, FC strength was negatively associated with the severity of subclinical depressive symptoms in the healthy participants in our study, which was different from the previous study that showed positive or negative correlations [30]. The reasons for these divergences probably were that we excluded subjects with alcohol abuse, alcohol dependence, marijuana dependence or positive psychoactive substance test results, which might interfere with brain functional activity. Brain functional features altered differently in various mental conditions. One study found that radiomics analyses could accurately distinguish subclinical depression from MDD based on different brain alterations [83], and another systematic review found that alterations in brain structure and functional activity in individuals with MDD, ANX or their comorbidity had their features [84]. FCs associated with one kind of symptoms are likely to differ when individuals are in different mental health conditions, and thus it is necessary to consider the differences to relieve them.

## Limitations

Our study presents several limitations. First, three items, including feeling tired, heart pounding and sleep problems, overlap across the three subscales [40], which may have caused the results to deviate from reality. If the items in one subscale that overlap with those in the other two subscales are removed, the results may become more specific, but the power to reflect the full domain of symptoms may become weaker. Second, we did not include participants without subclinical symptoms. Therefore, we cannot identify whether these FCs associated with the symptoms are abnormal. Third, the number of subjects is relatively small in our study, and thus it should be cautious to generalize our findings to other populations. Fourth, we cannot obtain treatment information and other clinical information, which may alter brain activities [85]. It should be validated in a larger sample size and more comprehensive population in the future.

## Conclusions

Somatic symptoms partially mediate the interaction between subclinical anxiety and depressive symptoms. The FC between the right medial superior frontal gyrus and the left thalamus in healthy people and the common FCs mainly involving the triangular part in the left inferior frontal gyrus, bilateral postcentral gyri, bilateral insula, bilateral precentral gyri and bilateral cingulate gyri in remission of MDD maybe underlie the mediating effect of somatic symptoms on the association between subclinical anxiety and depressive symptoms. Our findings may contribute to a better understanding of these symptoms, and the common FCs may be potential targets for intervention to prevent the onset/relapse of ANX/MDD.

## Supplementary Information

**Additional file 1.**

## Data Availability

Data in this study are obtained from HCP and available from the website: http://www.humanconnectome.org.

## Abbreviations

HP: Healthy participants
MDP: Participants in remission of major depressive disorder
ANX: Anxiety disorders
MDD: Major depressive disorder
FCs: Functional connectivities
HCP: Human Connectome Project
rs-fMRI: Resting-state functional magnetic resonance imaging
SSAGA: Semi-Structured Assessment for the Genetics of Alcoholism
DSM-IV: Diagnostic and Statistical Manual of Mental Disorders, Fourth Edition
ASR: Achenbach Adult Self-Report
FSL: FMRIB Software Library
CI: Confidence interval
FDR: False discovery rate
AS-FC: The common functional connectivities of subclinical anxiety and somatic symptoms
DS-FC: The common functional connectivities of subclinical depressive and somatic symptoms.
