# Supplementary material for: Somatic symptoms mediate the association between subclinical anxiety and depressive symptoms and its neuroimaging mechanisms
Source: BMC Psychiatry. 2022 Dec 29;22:835. doi: 10.1186/s12888-022-04488-9 (PMC9798660; doi:10.1186/s12888-022-04488-9)
Supplement: Supplementary file 1 — Additional file 1. [file 12888_2022_4488_MOESM1_ESM.docx]

Table S1 The items of the DSM-Ⅳ Anxiety Problems subscale, DSM-IV Depressive Problems subscale and Somatic Complaints subscale

| DSM-IV Anxiety Problems | DSM-IV Depressive Problems | Somatic Complaints |
| --- | --- | --- |
| 22. I worry about my future | 14. I cry a lot | 51. I feel dizzy or lightheaded |
| 29. I am afraid of certain animals, situations, or places | 18. I deliberately try to hurt or kill myself | 54. I feel tired without good reason |
| 45. I am nervous or tense | 24. I don't eat as well as I should | 56a. Aches or pains (not stomach or headaches) |
| 50. I am too fearful or anxious | 35. I feel worthless or inferior | 56b. Headaches |
| 56h. Heart pounding or racing | 52. I feel too guilty | 56c. Nausea, feel sick |
| 72. I worry about my family | 54. I feel tired without good reason | 56d. Problems with eyes (not if corrected by glasses) |
| 112. I worry a lot | 60. There is very little that I enjoy | 56e. Rashes or other skin problems |
|  | 77. I sleep more than most other people during day and/or night | 56f. Stomach aches |
|  | 78. I have trouble making decisions | 56g. Vomiting, throwing up |
|  | 91. I think about killing myself | 56h. Heart pounding or racing |
|  | 100. I have trouble sleeping | 56i. Numbness or tingling in body parts |
|  | 102. I don't have much energy | 100. I have trouble sleeping |
|  | 103. I am unhappy, sad, or depressed |  |
|  | 107. I feel that I can't succeed |  |

Note: The DSM-Ⅳ Anxiety Problems subscale, DSM-IV Depressive Problems subscale and Somatic Complaints subscale are subscales of the Achenbach Adult Self-Report (ASR) for ages 18-59.

Table S2 Correlations between each two of anxiety, depressive and somatic complaints score

| Spearman partial correlation | HP | | | MDP | | |
| --- | --- | --- | --- | --- | --- | --- |
|  | Anxiety score | Depressive score | Somatic complaints score | Anxiety score | Depressive score | Somatic complaints score |
| Anxiety score | 1 | - | - | 1 | - | - |
| Depressive score | 0.557^***^ | 1 | - | 0.639^***^ | 1 | - |
| Somatic complaints score | 0.451^***^ | 0.604^***^ | 1 | 0.601^***^ | 0.612^***^ | 1 |

^***^*p* < 0.001; HP: healthy participants; MDP: participants in remission of major depressive disorder.

Table S3 The functional connectivities associated with subclinical anxiety symptoms in healthy participants

| Functional connectivities | | Coefficient | t-value | *p*-value-FDR |
| --- | --- | --- | --- | --- |
| Region 1 | Region 2 |  |  |  |
| 87: Right superior frontal gyrus, dorsolateral | 232: Left middle cingulate & paracingulate gyri | –1.4752 | –3.5150 | 0.0006 |
| 93: Right middle cingulate & paracingulate gyri | 172: Left supplementary motor area | –1.9025 | –4.0863 | 0.0002 |
| 101: Right inferior parietal gyrus, excluding  supramarginal and angular gyri | 232: Left middle cingulate & paracingulate gyri | –1.5973 | –3.4487 | 0.0007 |
| 107: Right inferior frontal gyrus, triangular part | 234: Left thalamus | –1.9129 | –3.5875 | 0.0006 |
| 130: Right posterior cingulate gyrus | 186: Left posterior cingulate gyrus | –1.4733 | –3.2479 | 0.0012 |
| 172: Left supplementary motor area | 232: Left middle cingulate & paracingulate gyri | –1.9990 | –4.1204 | 0.0002 |
| 224: Left superior frontal gyrus, medial | 232: Left middle cingulate & paracingulate gyri | –1.7695 | –3.7286 | 0.0005 |
| 232: Left middle cingulate & paracingulate gyri | 243: Left calcarine fissure and surrounding  cortex | –2.1406 | –3.9936 | 0.0002 |
| 232: Left middle cingulate & paracingulate gyri | 247: Left middle occipital gyrus | –1.8866 | –3.6751 | 0.0005 |

*p*-value-FDR: *p* value corrected by false discovery rate.

Table S4 The functional connectivities associated with subclinical depressive symptoms in healthy participants

| Functional connectivities | | coefficient | t-value | *p*-value-FDR |
| --- | --- | --- | --- | --- |
| Region 1 | Region 2 |  |  |  |
| 8: Left rectus gyrus | 209: Left superior parietal gyrus | –2.5457 | –3.7056 | 0.0005 |
| 22: Right postcentral gyrus | 58: Right postcentral gyrus | –2.2637 | –3.6218 | 0.0006 |
| 23: Right middle frontal gyrus | 134: Right precentral gyrus | –1.7059 | –3.2938 | 0.0012 |
| 55: Right superior frontal gyrus, medial | 234: Left thalamus | –2.3485 | –3.8286 | 0.0004 |
| 70: Right middle frontal gyrus | 230: Left precuneus | –1.8033 | –3.1056 | 0.0020 |
| 130: Right posterior cingulate gyrus | 186: Left posterior cingulate gyrus | –2.3020 | –3.8325 | 0.0004 |
| 139: Left inferior temporal gyrus | 186: Left posterior cingulate gyrus | –2.6937 | –3.3843 | 0.0011 |
| 155: Left thalamus | 185: Left superior frontal gyrus, dorsolateral | –2.5982 | –4.1598 | 0.0003 |
| 162: Left precentral gyrus | 249: Left supplementary motor area | –2.0007 | –3.3550 | 0.0011 |

*p*-value-FDR: *p* value corrected by false discovery rate.

Table S5 The functional connectivities associated with somatic symptoms in healthy participants

| Functional connectivities | | coefficient | t-value | *p*-value-FDR |
| --- | --- | --- | --- | --- |
| Region 1 | Region 2 |  |  |  |
| 4: Left anterior orbitofrontal gyrus | 22: Right postcentral gyrus | –1.8207 | –3.7134 | 0.0006 |
| 6: Left medial orbitofrontal gyrus | 44: Right postcentral gyrus | –2.3032 | –3.9858 | 0.0006 |
| 6: Left medial orbitofrontal gyrus | 65: Right postcentral gyrus | –1.8789 | –3.6481 | 0.0006 |
| 11: Right lateral orbitofrontal gyrus | 81: Right temporal pole: superior temporal gyrus | –1.5231 | –3.2843 | 0.0012 |
| 12: Left lateral orbitofrontal gyrus | 65: Right postcentral gyrus | –2.3412 | –3.8530 | 0.0006 |
| 14: Left inferior frontal gyrus, orbital part | 228: Left paracentral lobule | –1.9230 | –3.6620 | 0.0006 |
| 18: Right postcentral gyrus | 162: Left precentral gyrus | –1.9649 | –3.6272 | 0.0006 |
| 19: Right supramarginal gyrus | 23: Right middle frontal gyrus | –2.0258 | –4.0544 | 0.0006 |
| 19: Right supramarginal gyrus | 244: Left anterior cingulate & paracingulate  gyri | –2.0526 | –3.5388 | 0.0006 |
| 20: Lateral orbitofrontal cortex | 210: Left hippocampus | –2.0204 | –3.5474 | 0.0006 |
| 22: Right postcentral gyrus | 135: Left middle frontal gyrus | –1.6538 | –3.2527 | 0.0013 |
| 22: Right postcentral gyrus | 162: Left precentral gyrus | –1.8875 | –3.6324 | 0.0006 |
| 22: Right postcentral gyrus | 177: Right lateral orbital gyrus | –1.6112 | –3.6610 | 0.0006 |
| 23: Right middle frontal gyrus | 44: Right postcentral gyrus | –2.0989 | –3.8881 | 0.0006 |
| 40: Right postcentral gyrus | 188: Left precentral gyrus | –1.6131 | –3.1996 | 0.0015 |
| 40: Right postcentral gyrus | 211: Left middle temporal gyrus | –1.8263 | –3.5177 | 0.0007 |
| 41: Right superior parietal gyrus | 167: Left anterior cingulate & paracingulate gyri | –1.8250 | –3.8045 | 0.0006 |
| 41: Right superior parietal gyrus | 233: Left middle frontal gyrus | –1.6770 | –3.4578 | 0.0007 |
| 42: Right postcentral gyrus | 188: Left precentral gyrus | –1.7771 | –3.4555 | 0.0007 |
| 46: Right cuneus | 57: Right calcarine fissure and surrounding cortex | –1.7358 | –3.4956 | 0.0007 |

Table S5 The functional connectivities associated with somatic symptoms in healthy participants (continued)

| Functional connectivities | | coefficient | t-value | *p*-value-FDR |
| --- | --- | --- | --- | --- |
| Region 1 | Region 2 |  |  |  |
| 46: Right cuneus | 158: Left precuneus | –1.9295 | –3.7452 | 0.0006 |
| 46: Right cuneus | 226: Left middle occipital gyrus | –2.0580 | –3.6702 | 0.0006 |
| 49: Right superior occipital gyrus | 226: Left middle occipital gyrus | –1.9567 | –3.5795 | 0.0006 |
| 55: Right superior frontal gyrus, medial | 234: Left thalamus | –2.2746 | –4.3898 | 0.0005 |
| 65: Right postcentral gyrus | 137: Left inferior frontal gyrus, opercular part | –2.2873 | –3.8584 | 0.0006 |
| 65: Right postcentral gyrus | 177: Right lateral orbital gyrus | –1.7381 | –3.6105 | 0.0006 |
| 65: Right postcentral gyrus | 188: Left precentral gyrus | –2.1713 | –3.9359 | 0.0006 |
| 72: Right superior frontal gyrus, medial orbital (or ventromedial prefrontal cortex) | 234: Left thalamus | –1.7153 | –3.2098 | 0.0015 |
| 95: Right inferior parietal gyrus, excluding supramarginal and angular gyri | 102: Right superior frontal gyrus, dorsolateral | –1.9392 | –3.4484 | 0.0007 |
| 107: Right inferior frontal gyrus, triangular part | 244: Left anterior cingulate & paracingulate gyri | –2.0946 | –3.7772 | 0.0006 |
| 123: Right inferior parietal gyrus, excluding  supramarginal and angular gyri | 149: Left inferior parietal gyrus, excluding supramarginal and angular gyri | –1.6183 | –3.2981 | 0.0012 |
| 127: Right superior frontal gyrus, medial | 186: Left posterior cingulate gyrus | –1.8501 | –3.5674 | 0.0006 |

*p*-value-FDR: *p* value corrected by false discovery rate.

Table S6 The functional connectivities associated with subclinical anxiety symptoms in remission of MDD

| Functional connectivities | | coefficient | t-value | *p*-value-FDR |
| --- | --- | --- | --- | --- |
| Region 1 | Region 2 |  |  |  |
| 1: Right posterior orbitofrontal gyrus | 173: Left temporal pole: superior temporal gyrus | 5.5407 | 3.3794 | 0.0017 |
| 1: Right posterior orbitofrontal gyrus | 247: Left middle occipital gyrus | 6.6991 | 3.4217 | 0.0015 |
| 2: Left posterior orbitofrontal gyrus | 72: Right superior frontal gyrus, medial orbital (or ventromedial prefrontal cortex) | 6.1005 | 4.3114 | 0.0006 |
| 2: Left posterior orbitofrontal gyrus | 125: Right lenticular nucleus, putamen | 5.3595 | 3.7486 | 0.0009 |
| 6: Left medial orbitofrontal gyrus | 145: Left postcentral gyrus | 8.5527 | 3.4344 | 0.0015 |
| 6: Left medial orbitofrontal gyrus | 166: Left temporal pole: middle temporal gyrus | 4.8561 | 3.9706 | 0.0007 |
| 7: Right gyrus rectus | 149: Left inferior parietal gyrus, excluding supramarginal and angular gyri | 6.6218 | 4.1050 | 0.0006 |
| 7: Right gyrus rectus | 217: Left supramarginal gyrus | 6.5350 | 3.4272 | 0.0015 |
| 8: Left gyrus rectus | 72: Right superior frontal gyrus, medial orbital (or ventromedial prefrontal cortex) | 4.3851 | 3.4844 | 0.0014 |
| 8: Left gyrus rectus | 151: Left insula | 5.1246 | 3.5426 | 0.0012 |
| 9: Right olfactory cortex | 178: Left angular gyrus | 7.6525 | 4.1115 | 0.0006 |
| 10: Left olfactory cortex | 178: Left angular gyrus | 8.3961 | 4.3699 | 0.0006 |
| 10: Left olfactory cortex | 199: Left inferior parietal gyrus, excluding supramarginal and angular gyri | 7.0383 | 3.7244 | 0.0009 |
| 10: Left olfactory cortex | 220: Left temporal pole: superior temporal gyrus | 5.7669 | 3.8286 | 0.0008 |
| 12: Left lateral orbitofrontal gyrus | 249: Left supplementary motor area | 5.8842 | 3.5618 | 0.0012 |
| 16: Right superior frontal gyrus, dorsolateral | 162: Left precentral gyrus | 5.5512 | 4.0636 | 0.0007 |

Table S6 The functional connectivities associated with subclinical anxiety symptoms in remission of MDD (continued)

| Functional connectivities | | coefficient | t-value | *p*-value-FDR |
| --- | --- | --- | --- | --- |
| Region 1 | Region 2 |  |  |  |
| 16: Right superior frontal gyrus, dorsolateral | 184: Left inferior temporal gyrus | 6.4475 | 4.1258 | 0.0006 |
| 16: Right superior frontal gyrus, dorsolateral | 214: Left superior temporal gyrus | 5.1578 | 3.4746 | 0.0014 |
| 21: Right precentral gyrus | 180: Left precentral gyrus | 4.4243 | 3.5022 | 0.0013 |
| 25: Right thalamus | 215: Left inferior temporal gyrus | 6.4435 | 3.6457 | 0.0010 |
| 28: Right heschl's gyrus | 185: Left superior frontal gyrus, dorsolateral | 5.7143 | 3.3467 | 0.0018 |
| 33: Right inferior frontal gyrus, triangular part | 88: Right fusiform gyrus | 6.4533 | 3.6923 | 0.0009 |
| 37: Right caudate nucleus | 162: Left precentral gyrus | 5.9894 | 3.8743 | 0.0008 |
| 37: Right caudate nucleus | 227: Left middle frontal gyrus | 6.2601 | 4.2355 | 0.0006 |
| 37: Right caudate nucleus | 237: Left middle cingulate & paracingulate gyri | 5.3192 | 3.6705 | 0.0010 |
| 38: Right temporal pole: middle temporal gyrus | 148: Left postcentral gyrus | 5.6620 | 3.7029 | 0.0009 |
| 38: Right temporal pole: middle temporal gyrus | 149: Left inferior parietal gyrus, excluding supramarginal and angular gyri | 6.7573 | 4.4727 | 0.0006 |
| 38: Right temporal pole: middle temporal gyrus | 168: Left superior temporal gyrus | 5.5463 | 4.0225 | 0.0007 |
| 38: Right temporal pole: middle temporal gyrus | 171: Left precuneus | 4.9684 | 3.7820 | 0.0009 |
| 38: Right temporal pole: middle temporal gyrus | 175: Left hippocampus | 4.8643 | 3.6577 | 0.0010 |
| 38: Right temporal pole: middle temporal gyrus | 179: Left supramarginal gyrus | 5.3207 | 3.7525 | 0.0009 |
| 38: Right temporal pole: middle temporal gyrus | 187: Left lingual gyrus | 5.2319 | 3.6043 | 0.0011 |
| 38: Right temporal pole: middle temporal gyrus | 189: Left insula | 5.1099 | 3.8959 | 0.0008 |
| 38: Right temporal pole: middle temporal gyrus | 190: Left lingual gyrus | 4.4631 | 3.5028 | 0.0013 |
| 38: Right temporal pole: middle temporal gyrus | 196: Left insula | 4.6891 | 3.6894 | 0.0009 |

Table S6 The functional connectivities associated with subclinical anxiety symptoms in remission of MDD (continued)

| Functional connectivities | | coefficient | t-value | *p*-value-FDR |
| --- | --- | --- | --- | --- |
| Region 1 | Region 2 |  |  |  |
| 38: Right temporal pole: middle temporal gyrus | 198: Left superior temporal gyrus | 5.0481 | 3.6834 | 0.0009 |
| 38: Right temporal pole: middle temporal gyrus | 199: Left inferior parietal gyrus, excluding supramarginal and angular gyri | 5.4031 | 3.8964 | 0.0008 |
| 38: Right temporal pole: middle temporal gyrus | 214: Left superior temporal gyrus | 5.5288 | 4.1024 | 0.0006 |
| 38: Right temporal pole: middle temporal gyrus | 215: Left inferior temporal gyrus | 5.7221 | 4.0287 | 0.0007 |
| 38: Right temporal pole: middle temporal gyrus | 222: Left insula | 5.6322 | 4.1781 | 0.0006 |
| 39: Right middle cingulate & paracingulate gyri | 65: Right postcentral gyrus | 6.0397 | 4.0028 | 0.0007 |
| 39: Right middle cingulate & paracingulate gyri | 81: Right temporal pole: superior temporal gyrus | 5.5675 | 3.8281 | 0.0008 |
| 39: Right middle cingulate & paracingulate gyri | 206: Left superior frontal gyrus, dorsolateral | 4.3470 | 3.5736 | 0.0011 |
| 39: Right middle cingulate & paracingulate gyri | 222: Left insula | 6.6240 | 5.4112 | 0.0003 |
| 47: Right superior frontal gyrus, medial | 151: Left insula | 5.0770 | 3.8654 | 0.0008 |
| 50: Right precuneus | 58: Right postcentral gyrus | 8.0390 | 4.0285 | 0.0007 |
| 50: Right precuneus | 220: Left temporal pole: superior temporal gyrus | 6.2919 | 3.9914 | 0.0007 |
| 57: Right calcarine fissure and surrounding cortex | 91: Right inferior temporal gyrus | 5.2671 | 3.2704 | 0.0022 |
| 59: Right middle temporal gyrus | 184: Left inferior temporal gyrus | 6.2260 | 3.7500 | 0.0009 |
| 60: Right caudate nucleus | 162: Left precentral gyrus | 8.4578 | 5.3970 | 0.0003 |
| 62: Right anterior cingulate & paracingulate gyri | 159: Left supplementary motor area | 5.1620 | 3.7817 | 0.0009 |
| 62: Right anterior cingulate & paracingulate gyri | 162: Left precentral gyrus | 5.8082 | 4.6444 | 0.0006 |
| 62: Right anterior cingulate & paracingulate gyri | 178: Left angular gyrus | 7.0243 | 4.2307 | 0.0006 |
| 62: Right anterior cingulate & paracingulate gyri | 180: Left precentral gyrus | 5.4560 | 3.7544 | 0.0009 |

Table S6 The functional connectivities associated with subclinical anxiety symptoms in remission of MDD (continued)

| Functional connectivities | | coefficient | t-value | *p*-value-FDR |
| --- | --- | --- | --- | --- |
| Region 1 | Region 2 |  |  |  |
| 62: Right anterior cingulate & paracingulate gyri | 227: Left middle frontal gyrus | 4.8750 | 3.6027 | 0.0011 |
| 63: Right middle cingulate & paracingulate gyri | 184: Left inferior temporal gyrus | 6.7388 | 4.4878 | 0.0006 |
| 63: Right middle cingulate & paracingulate gyri | 214: Left superior temporal gyrus | 5.0450 | 3.7320 | 0.0009 |
| 64: Right superior temporal gyrus | 158: Left precuneus | 5.6182 | 3.4634 | 0.0014 |
| 64: Right superior temporal gyrus | 197: Left fusiform gyrus | 7.5019 | 3.8485 | 0.0008 |
| 66: Right hippocampus | 181: Left inferior temporal gyrus | 6.6323 | 3.6725 | 0.0010 |
| 67: Right supplementary motor area | 206: Left superior frontal gyrus, dorsolateral | 4.9817 | 4.0417 | 0.0007 |
| 69: Right middle temporal gyrus | 197: Left fusiform gyrus | 5.9905 | 3.7574 | 0.0009 |
| 72: Right superior frontal gyrus, medial orbital (or ventromedial prefrontal cortex) | 149: Left inferior parietal gyrus, excluding supramarginal and angular gyri | 6.3681 | 3.9263 | 0.0008 |
| 72: Right superior frontal gyrus, medial orbital (or ventromedial prefrontal cortex) | 151: Left insula | 6.1239 | 4.2701 | 0.0006 |
| 73: Right middle frontal gyrus | 184: Left inferior temporal gyrus | 5.2817 | 3.3792 | 0.0017 |
| 73: Right middle frontal gyrus | 188: Left precentral gyrus | 5.2261 | 3.6849 | 0.0009 |
| 74: Right anterior cingulate & paracingulate gyri | 137: Left inferior frontal gyrus, opercular part | 4.9617 | 3.5558 | 0.0012 |
| 74: Right anterior cingulate & paracingulate gyri | 162: Left precentral gyrus | 5.2930 | 4.5143 | 0.0006 |
| 74: Right anterior cingulate & paracingulate gyri | 168: Left superior temporal gyrus | 6.9951 | 3.6153 | 0.0011 |
| 74: Right anterior cingulate & paracingulate gyri | 172: Left supplementary motor area | 4.1612 | 3.4300 | 0.0015 |
| 74: Right anterior cingulate & paracingulate gyri | 178: Left angular gyrus | 7.3533 | 4.8783 | 0.0006 |
| 74: Right anterior cingulate & paracingulate gyri | 179: Left supramarginal gyrus | 5.8737 | 3.8770 | 0.0008 |

Table S6 The functional connectivities associated with subclinical anxiety symptoms in remission of MDD (continued)

| Functional connectivities | | coefficient | t-value | *p*-value-FDR |
| --- | --- | --- | --- | --- |
| Region 1 | Region 2 |  |  |  |
| 74: Right anterior cingulate & paracingulate gyri | 198: Left superior temporal gyrus | 6.1270 | 4.2861 | 0.0006 |
| 74: Right anterior cingulate & paracingulate gyri | 199: Left inferior parietal gyrus, excluding supramarginal and angular gyri | 5.8158 | 4.0032 | 0.0007 |
| 74: Right anterior cingulate & paracingulate gyri | 214: Left superior temporal gyrus | 5.6704 | 4.3651 | 0.0006 |
| 74: Right anterior cingulate & paracingulate gyri | 222: Left insula | 6.8213 | 4.5455 | 0.0006 |
| 74: Right anterior cingulate & paracingulate gyri | 229: Left postcentral gyrus | 5.9315 | 3.5967 | 0.0011 |
| 75: Right temporal pole: middle temporal gyrus | 151: Left insula | 6.3606 | 4.0754 | 0.0007 |
| 75: Right temporal pole: middle temporal gyrus | 168: Left superior temporal gyrus | 4.9095 | 3.9048 | 0.0008 |
| 75: Right temporal pole: middle temporal gyrus | 189: Left insula | 5.6826 | 4.1059 | 0.0006 |
| 75: Right temporal pole: middle temporal gyrus | 204: Left middle frontal gyrus | 6.5046 | 3.8812 | 0.0008 |
| 75: Right temporal pole: middle temporal gyrus | 215: Left inferior temporal gyrus | 5.5658 | 3.7606 | 0.0009 |
| 76: Right middle cingulate & paracingulate gyri | 222: Left insula | 6.1609 | 4.6226 | 0.0006 |
| 81: Right temporal pole: superior temporal gyrus | 222: Left insula | 5.5071 | 3.6515 | 0.0010 |
| 82: Right superior parietal gyrus | 178: Left angular gyrus | 5.7118 | 3.5595 | 0.0012 |
| 82: Right superior parietal gyrus | 220: Left temporal pole: superior temporal gyrus | 5.8155 | 3.6041 | 0.0011 |
| 83: Right anterior cingulate & paracingulate gyri | 151: Left insula | 5.1574 | 3.4229 | 0.0015 |
| 83: Right anterior cingulate & paracingulate gyri | 162: Left precentral gyrus | 4.5992 | 3.7517 | 0.0009 |
| 83: Right anterior cingulate & paracingulate gyri | 178: Left angular gyrus | 7.1912 | 4.6585 | 0.0006 |
| 83: Right anterior cingulate & paracingulate gyri | 222: Left insula | 4.9680 | 3.3563 | 0.0018 |
| 84: Right insula | 164: Left middle frontal gyrus | 5.8785 | 3.4332 | 0.0015 |

Table S6 The functional connectivities associated with subclinical anxiety symptoms in remission of MDD (continued)

| Functional connectivities | | coefficient | t-value | *p*-value-FDR |
| --- | --- | --- | --- | --- |
| Region 1 | Region 2 |  |  |  |
| 84: Right insula | 173: Left temporal pole: superior temporal gyrus | 4.9448 | 3.5456 | 0.0012 |
| 84: Right insula | 212: Left fusiform gyrus | 5.4920 | 3.3335 | 0.0019 |
| 89: Right middle frontal gyrus | 151: Left insula | 6.4517 | 3.7025 | 0.0009 |
| 89: Right middle frontal gyrus | 227: Left middle frontal gyrus | 5.0809 | 3.8900 | 0.0008 |
| 89: Right middle frontal gyrus | 240: Left superior frontal gyrus, medial | 4.5228 | 3.5127 | 0.0013 |
| 90: Right middle temporal gyrus | 149: Left inferior parietal gyrus, excluding supramarginal and angular gyri | 6.6202 | 3.9442 | 0.0008 |
| 90: Right middle temporal gyrus | 222: Left insula | 4.6584 | 3.5099 | 0.0013 |
| 92: Right insula | 151: Left insula | 6.8482 | 3.9239 | 0.0008 |
| 92: Right insula | 222: Left insula | 7.3581 | 4.1126 | 0.0006 |
| 98: Right fusiform gyrus | 109: Right fusiform gyrus | 7.0585 | 3.7938 | 0.0009 |
| 102: Right superior frontal gyrus, dorsolateral | 222: Left insula | 5.9191 | 4.3105 | 0.0006 |
| 104: Right inferior frontal gyrus, triangular part | 158: Left precuneus | 6.0056 | 3.3222 | 0.0019 |
| 105: Right supplementary motor area | 162: Left precentral gyrus | 6.9882 | 4.5328 | 0.0006 |
| 105: Right supplementary motor area | 179: Left supramarginal gyrus | 6.9799 | 4.1521 | 0.0006 |
| 105: Right supplementary motor area | 180: Left precentral gyrus | 5.7224 | 4.2344 | 0.0006 |
| 107: Right inferior frontal gyrus, triangular part | 109: Right fusiform gyrus | 7.7097 | 4.1171 | 0.0006 |
| 117: Right superior frontal gyrus, medial orbital (or ventromedial prefrontal cortex) | 145: Left postcentral gyrus | 8.5808 | 3.8061 | 0.0009 |

Table S6 The functional connectivities associated with subclinical anxiety symptoms in remission of MDD (continued)

| Functional connectivities | | coefficient | t-value | *p*-value-FDR |
| --- | --- | --- | --- | --- |
| Region 1 | Region 2 |  |  |  |
| 117: Right superior frontal gyrus, medial orbital (or ventromedial prefrontal cortex) | 160: Left middle cingulate & paracingulate gyri | 6.1216 | 3.7750 | 0.0009 |
| 117: Right superior frontal gyrus, medial orbital (or ventromedial prefrontal cortex) | 180: Left precentral gyrus | 7.6981 | 4.3303 | 0.0006 |
| 117: Right superior frontal gyrus, medial orbital (or ventromedial prefrontal cortex) | 185: Left superior frontal gyrus, dorsolateral | 6.0077 | 4.3131 | 0.0006 |
| 117: Right superior frontal gyrus, medial orbital (or ventromedial prefrontal cortex) | 198: Left superior temporal gyrus | 6.6761 | 3.6432 | 0.0010 |
| 117: Right superior frontal gyrus, medial orbital (or ventromedial prefrontal cortex) | 229: Left postcentral gyrus | 6.9296 | 3.7699 | 0.0009 |
| 121: Right temporal pole: middle temporal gyrus | 168: Left superior temporal gyrus | 4.5984 | 4.0275 | 0.0007 |
| 121: Right temporal pole: middle temporal gyrus | 184: Left inferior temporal gyrus | 5.6333 | 3.4362 | 0.0015 |
| 121: Right temporal pole: middle temporal gyrus | 189: Left insula | 5.5015 | 4.2805 | 0.0006 |
| 121: Right temporal pole: middle temporal gyrus | 215: Left inferior temporal gyrus | 5.2272 | 3.4884 | 0.0013 |
| 121: Right temporal pole: middle temporal gyrus | 222: Left insula | 5.2961 | 4.6293 | 0.0006 |
| 125: Right lenticular nucleus, putamen | 151: Left insula | 6.4857 | 4.5154 | 0.0006 |
| 125: Right lenticular nucleus, putamen | 178: Left angular gyrus | 7.3679 | 3.6334 | 0.0010 |
| 127: Right superior frontal gyrus, medial | 151: Left insula | 5.7635 | 3.9170 | 0.0008 |
| 127: Right superior frontal gyrus, medial | 178: Left angular gyrus | 6.9781 | 4.2040 | 0.0006 |
| 128: Right cuneus | 184: Left inferior temporal gyrus | 5.5984 | 3.2885 | 0.0021 |
| 129: Right parahippocampal gyrus | 168: Left superior temporal gyrus | 6.2710 | 4.3161 | 0.0006 |

Table S6 The functional connectivities associated with subclinical anxiety symptoms in remission of MDD (continued)

| Functional connectivities | | coefficient | t-value | *p*-value-FDR |
| --- | --- | --- | --- | --- |
| Region 1 | Region 2 |  |  |  |
| 129: Right parahippocampal gyrus | 187: Left lingual gyrus | 6.1159 | 3.9306 | 0.0008 |
| 129: Right parahippocampal gyrus | 189: Left insula | 5.1754 | 3.7333 | 0.0009 |
| 129: Right parahippocampal gyrus | 222: Left insula | 5.0041 | 3.7922 | 0.0009 |
| 132: Right middle frontal gyrus | 151: Left insula | 6.0646 | 3.7287 | 0.0009 |
| 132: Right middle frontal gyrus | 222: Left insula | 7.2831 | 4.2105 | 0.0006 |
| 135: Left middle frontal gyrus | 151: Left insula | 5.7285 | 3.7353 | 0.0009 |
| 135: Left middle frontal gyrus | 233: Left middle frontal gyrus | 4.5607 | 4.1044 | 0.0006 |
| 135: Left middle frontal gyrus | 239: Left hippocampus | 7.1256 | 4.1500 | 0.0006 |
| 136: Left inferior frontal gyrus, triangular part | 224: Left superior frontal gyrus, medial | 5.4420 | 3.9293 | 0.0008 |
| 138: Left precuneus | 151: Left insula | 8.7393 | 3.9819 | 0.0007 |
| 142: Left caudate nucleus | 227: Left middle frontal gyrus | 6.2388 | 3.7161 | 0.0009 |
| 145: Left postcentral gyrus | 179: Left supramarginal gyrus | 7.6719 | 4.1134 | 0.0006 |
| 146: Left thalamus | 227: Left middle frontal gyrus | 7.2879 | 3.9993 | 0.0007 |
| 150: Left anterior cingulate & paracingulate gyri | 222: Left insula | 7.5426 | 4.0263 | 0.0007 |
| 151: Left insula | 167: Left anterior cingulate & paracingulate gyri | 6.7755 | 4.5244 | 0.0006 |
| 151: Left insula | 178: Left angular gyrus | 9.2727 | 3.7028 | 0.0009 |
| 151: Left insula | 183: Left superior frontal gyrus, dorsolateral | 6.0944 | 4.1804 | 0.0006 |
| 151: Left insula | 203: Left superior frontal gyrus, medial | 6.0161 | 4.1741 | 0.0006 |
| 151: Left insula | 236: Left caudate nucleus | 7.2478 | 4.5950 | 0.0006 |
| 151: Left insula | 237: Left middle cingulate & paracingulate gyri | 5.4741 | 3.6343 | 0.0010 |

Table S6 The functional connectivities associated with subclinical anxiety symptoms in remission of MDD (continued)

| Functional connectivities | | coefficient | t-value | *p*-value-FDR |
| --- | --- | --- | --- | --- |
| Region 1 | Region 2 |  |  |  |
| 151: Left insula | 240: Left superior frontal gyrus, medial | 7.2786 | 5.1270 | 0.0004 |
| 151: Left insula | 245: Left caudate nucleus | 6.4383 | 4.1765 | 0.0006 |
| 160: Left middle cingulate & paracingulate gyri | 240: Left superior frontal gyrus, medial | 4.7355 | 3.7780 | 0.0009 |
| 160: Left middle cingulate & paracingulate gyri | 244: Left anterior cingulate & paracingulate gyri | 5.2632 | 3.6110 | 0.0011 |
| 161: Left middle cingulate & paracingulate gyri | 222: Left insula | 5.2204 | 3.5819 | 0.0011 |
| 166: Left temporal pole: middle temporal gyrus | 190: Left lingual gyrus | 5.0504 | 3.3431 | 0.0018 |
| 167: Left anterior cingulate & paracingulate gyri | 178: Left angular gyrus | 6.8374 | 4.1450 | 0.0006 |
| 167: Left anterior cingulate & paracingulate gyri | 199: Left inferior parietal gyrus, excluding supramarginal and angular gyri | 6.1597 | 3.7191 | 0.0009 |
| 167: Left anterior cingulate & paracingulate gyri | 240: Left superior frontal gyrus, medial | 5.1744 | 4.0462 | 0.0007 |
| 173: Left temporal pole: superior temporal gyrus | 190: Left lingual gyrus | 5.8349 | 3.8451 | 0.0008 |
| 173: Left temporal pole: superior temporal gyrus | 208: Left middle cingulate & paracingulate gyri | 6.2274 | 4.2065 | 0.0006 |
| 178: Left angular gyrus | 216: Left superior parietal gyrus | 6.7037 | 3.4811 | 0.0014 |
| 189: Left insula | 242: Left insula | 5.2074 | 3.8776 | 0.0008 |
| 190: Left lingual gyrus | 195: Left inferior frontal gyrus, triangular part | 6.1814 | 3.7801 | 0.0009 |
| 195: Left inferior frontal gyrus, triangular part | 229: Left postcentral gyrus | 6.4367 | 3.5417 | 0.0012 |
| 199: Left inferior parietal gyrus, excluding supramarginal and angular gyri | 203: Left superior frontal gyrus, medial | 5.5819 | 3.7086 | 0.0009 |
| 222: Left insula | 242: Left insula | 4.8121 | 3.9378 | 0.0008 |
| 227: Left middle frontal gyrus | 233: Left middle frontal gyrus | 5.3011 | 4.2947 | 0.0006 |

Table S6 The functional connectivities associated with subclinical anxiety symptoms in remission of MDD (continued)

| Functional connectivities | | coefficient | t-value | *p*-value-FDR |
| --- | --- | --- | --- | --- |
| Region 1 | Region 2 |  |  |  |
| 227: Left middle frontal gyrus | 240: Left superior frontal gyrus, medial | 4.4935 | 3.4728 | 0.0014 |
| 237: Left middle cingulate & paracingulate gyri | 244: Left anterior cingulate & paracingulate gyri | 5.4751 | 3.8660 | 0.0008 |
| 241: Left superior temporal gyrus | 242: Left insula | 6.4856 | 3.5431 | 0.0012 |

*p*-value-FDR: *p* value corrected by false discovery rate.

Table S7 The functional connectivities associated with subclinical depressive symptoms in remission of MDD

| Functional connectivities | | coefficient | t-value | *p*-value-FDR |
| --- | --- | --- | --- | --- |
| Region 1 | Region 2 |  |  |  |
| 6: Left medial orbitofrontal gyrus | 216: Left superior parietal gyrus | 13.5115 | 3.8088 | 0.0009 |
| 8: Left gyrus rectus | 72: Right superior frontal gyrus, medial orbital (or ventromedial prefrontal cortex) | 7.9425 | 3.9272 | 0.0008 |
| 9: Right olfactory cortex | 178: Left angular gyrus | 12.6103 | 4.0834 | 0.0008 |
| 10: Left olfactory cortex | 74: Right anterior cingulate & paracingulate gyri | 7.7892 | 3.7883 | 0.0009 |
| 10: Left olfactory cortex | 127: Right superior frontal gyrus, medial | 7.8261 | 3.9009 | 0.0008 |
| 10: Left olfactory cortex | 166: Left temporal pole: middle temporal gyrus | 7.6804 | 3.3118 | 0.0020 |
| 12: Left lateral orbitofrontal gyrus | 145: Left postcentral gyrus | 11.7945 | 3.6165 | 0.0011 |
| 12: Left lateral orbitofrontal gyrus | 146: Left thalamus | 12.0334 | 3.5306 | 0.0012 |
| 12: Left lateral orbitofrontal gyrus | 189: Left insula | 11.4535 | 3.7946 | 0.0009 |
| 12: Left lateral orbitofrontal gyrus | 190: Left lingual gyrus | 10.0042 | 3.9511 | 0.0008 |
| 16: Right superior frontal gyrus, dorsolateral | 188: Left precentral gyrus | 8.4061 | 3.6524 | 0.0010 |
| 16: Right superior frontal gyrus, dorsolateral | 250: Left postcentral gyrus | 8.6520 | 3.8713 | 0.0008 |
| 20: lateral orbitofrontal cortex | 173: Left temporal pole: superior temporal gyrus | 9.4224 | 3.3455 | 0.0018 |
| 24: Right inferior temporal gyrus | 171: Left precuneus | 7.7053 | 3.4883 | 0.0013 |
| 25: Right thalamus | 80: Right hippocampus | 8.4433 | 4.4327 | 0.0008 |
| 26: Right lingual gyrus | 226: Left middle occipital gyrus | 12.1640 | 4.2675 | 0.0008 |
| 28: Right heschl's gyrus | 160: Left middle cingulate & paracingulate gyri | 10.5152 | 4.4696 | 0.0008 |
| 28: Right heschl's gyrus | 172: Left supplementary motor area | 7.9409 | 3.5397 | 0.0012 |
| 28: Right heschl's gyrus | 185: Left superior frontal gyrus, dorsolateral | 9.1512 | 3.2085 | 0.0026 |
| 29: Right precuneus | 139: Left inferior temporal gyrus | 9.1578 | 3.3715 | 0.0017 |
| 31: Right superior temporal gyrus | 160: Left middle cingulate & paracingulate gyri | 7.8344 | 3.6378 | 0.0011 |
| 31: Right superior temporal gyrus | 194: Left middle occipital gyrus | 8.4167 | 3.5170 | 0.0013 |

Table S7 The functional connectivities associated with subclinical depressive symptoms in remission of MDD (continued)

| Functional connectivities | | coefficient | t-value | *p*-value-FDR |
| --- | --- | --- | --- | --- |
| Region 1 | Region 2 |  |  |  |
| 31: Right superior temporal gyrus | 209: Left superior parietal gyrus | 9.5940 | 3.8416 | 0.0008 |
| 31: Right superior temporal gyrus | 226: Left middle occipital gyrus | 9.4604 | 3.5028 | 0.0013 |
| 36: Right angular gyrus | 209: Left superior parietal gyrus | 10.2066 | 3.5761 | 0.0011 |
| 37: Right caudate nucleus | 125: Right lenticular nucleus, putamen | 8.5597 | 3.8505 | 0.0008 |
| 37: Right caudate nucleus | 127: Right superior frontal gyrus, medial | 8.1391 | 3.8510 | 0.0008 |
| 37: Right caudate nucleus | 178: Left angular gyrus | 12.5236 | 4.1105 | 0.0008 |
| 37: Right caudate nucleus | 180: Left precentral gyrus | 10.9178 | 3.7454 | 0.0010 |
| 38: Right temporal pole: middle temporal gyrus | 42: Right postcentral gyrus | 7.8740 | 3.8624 | 0.0008 |
| 38: Right temporal pole: middle temporal gyrus | 140: Left postcentral gyrus | 9.5495 | 4.3353 | 0.0008 |
| 38: Right temporal pole: middle temporal gyrus | 149: Left inferior parietal gyrus, excluding supramarginal and angular gyri | 10.2055 | 3.9217 | 0.0008 |
| 38: Right temporal pole: middle temporal gyrus | 171: Left precuneus | 10.0225 | 5.0537 | 0.0008 |
| 38: Right temporal pole: middle temporal gyrus | 174: Left cuneus | 11.3451 | 3.8405 | 0.0008 |
| 38: Right temporal pole: middle temporal gyrus | 176: Left angular gyrus | 11.5736 | 3.8657 | 0.0008 |
| 38: Right temporal pole: middle temporal gyrus | 178: Left angular gyrus | 12.2392 | 4.8940 | 0.0008 |
| 38: Right temporal pole: middle temporal gyrus | 180: Left precentral gyrus | 9.7747 | 3.8391 | 0.0008 |
| 38: Right temporal pole: middle temporal gyrus | 202: Left precuneus | 7.5071 | 3.8040 | 0.0009 |
| 38: Right temporal pole: middle temporal gyrus | 216: Left superior parietal gyrus | 9.9183 | 4.1651 | 0.0008 |
| 38: Right temporal pole: middle temporal gyrus | 226: Left middle occipital gyrus | 11.6131 | 4.5509 | 0.0008 |
| 39: Right middle cingulate & paracingulate gyri | 188: Left precentral gyrus | 11.8880 | 3.8686 | 0.0008 |
| 39: Right middle cingulate & paracingulate gyri | 206: Left superior frontal gyrus, dorsolateral | 7.2193 | 3.5874 | 0.0011 |
| 42: Right postcentral gyrus | 54: Right precuneus | 8.5131 | 3.6258 | 0.0011 |
| 43: Right middle temporal gyrus | 140: Left postcentral gyrus | 10.5690 | 3.8225 | 0.0009 |

Table S7 The functional connectivities associated with subclinical depressive symptoms in remission of MDD (continued)

| Functional connectivities | | coefficient | t-value | *p*-value-FDR |
| --- | --- | --- | --- | --- |
| Region 1 | Region 2 |  |  |  |
| 43: Right middle temporal gyrus | 171: Left precuneus | 10.9963 | 3.7805 | 0.0009 |
| 43: Right middle temporal gyrus | 202: Left precuneus | 11.2737 | 4.1615 | 0.0008 |
| 44: Right postcentral gyrus | 164: Left middle frontal gyrus | 10.9564 | 3.8722 | 0.0008 |
| 44: Right postcentral gyrus | 178: Left angular gyrus | 13.2413 | 4.3754 | 0.0008 |
| 44: Right postcentral gyrus | 209: Left superior parietal gyrus | 11.7485 | 3.6985 | 0.0010 |
| 45: Right inferior temporal gyrus | 184: Left inferior temporal gyrus | 8.5194 | 3.4106 | 0.0016 |
| 46: Right cuneus | 48: Right middle occipital gyrus | 11.1195 | 3.9535 | 0.0008 |
| 46: Right cuneus | 184: Left inferior temporal gyrus | 11.7480 | 3.6213 | 0.0011 |
| 47: Right superior frontal gyrus, medial | 125: Right lenticular nucleus, putamen | 8.2601 | 3.4531 | 0.0014 |
| 48: Right middle occipital gyrus | 184: Left inferior temporal gyrus | 12.4390 | 3.9887 | 0.0008 |
| 49: Right superior occipital gyrus | 58: Right postcentral gyrus | 12.3688 | 4.0605 | 0.0008 |
| 50: Right precuneus | 58: Right postcentral gyrus | 13.3422 | 4.0414 | 0.0008 |
| 50: Right precuneus | 166: Left temporal pole: middle temporal gyrus | 9.7388 | 3.6852 | 0.0010 |
| 52: Right insula | 142: Left caudate nucleus | 13.0757 | 3.7381 | 0.0010 |
| 53: Right superior temporal gyrus | 209: Left superior parietal gyrus | 9.9579 | 3.9533 | 0.0008 |
| 58: Right postcentral gyrus | 194: Left middle occipital gyrus | 9.8333 | 3.6182 | 0.0011 |
| 58: Right postcentral gyrus | 209: Left superior parietal gyrus | 14.4046 | 4.7387 | 0.0008 |
| 59: Right middle temporal gyrus | 171: Left precuneus | 11.6209 | 4.3351 | 0.0008 |
| 60: Right caudate nucleus | 134: Right precentral gyrus | 10.4006 | 3.9835 | 0.0008 |
| 60: Right caudate nucleus | 162: Left precentral gyrus | 12.1724 | 4.3296 | 0.0008 |
| 60: Right caudate nucleus | 172: Left supplementary motor area | 7.6874 | 3.3774 | 0.0017 |
| 60: Right caudate nucleus | 234: Left thalamus | 9.5899 | 3.3641 | 0.0017 |
| 62: Right anterior cingulate & paracingulate gyri | 125: Right lenticular nucleus, putamen | 8.5562 | 4.0929 | 0.0008 |

Table S7 The functional connectivities associated with subclinical depressive symptoms in remission of MDD (continued)

| Functional connectivities | | coefficient | t-value | *p*-value-FDR |
| --- | --- | --- | --- | --- |
| Region 1 | Region 2 |  |  |  |
| 63: Right middle cingulate & paracingulate gyri | 204: Left middle frontal gyrus | 9.6702 | 3.7142 | 0.0010 |
| 64: Right superior temporal gyrus | 173: Left temporal pole: superior temporal gyrus | 8.1612 | 4.0422 | 0.0008 |
| 64: Right superior temporal gyrus | 247: Left middle occipital gyrus | 9.0635 | 3.4468 | 0.0014 |
| 65: Right postcentral gyrus | 103: Right inferior occipital gyrus | 10.2117 | 3.6108 | 0.0011 |
| 66: Right hippocampus | 80: Right hippocampus | 11.9490 | 4.6003 | 0.0008 |
| 66: Right hippocampus | 186: Left posterior cingulate gyrus | 10.9454 | 3.4413 | 0.0014 |
| 67: Right supplementary motor area | 105: Right supplementary motor area | 10.1613 | 3.7029 | 0.0010 |
| 67: Right supplementary motor area | 143: Left middle cingulate & paracingulate gyri | 8.6461 | 3.4001 | 0.0016 |
| 70: Right middle frontal gyrus | 140: Left postcentral gyrus | 10.1943 | 3.3442 | 0.0018 |
| 72: Right superior frontal gyrus, medial orbital (or ventromedial prefrontal cortex) | 201: Left cuneus | 11.6230 | 3.6300 | 0.0011 |
| 74: Right anterior cingulate & paracingulate gyri | 178: Left angular gyrus | 10.9491 | 4.1615 | 0.0008 |
| 75: Right temporal pole: middle temporal gyrus | 140: Left postcentral gyrus | 10.8167 | 3.8701 | 0.0008 |
| 76: Right middle cingulate & paracingulate gyri | 180: Left precentral gyrus | 8.4403 | 3.8407 | 0.0008 |
| 77: Right middle frontal gyrus | 173: Left temporal pole: superior temporal gyrus | 12.5021 | 3.9729 | 0.0008 |
| 83: Right anterior cingulate & paracingulate gyri | 125: Right lenticular nucleus, putamen | 8.1480 | 3.5853 | 0.0011 |
| 92: Right insula | 127: Right superior frontal gyrus, medial | 6.9672 | 3.6659 | 0.0010 |
| 94: Right insula | 133: Right middle cingulate & paracingulate gyri | 10.9328 | 3.5357 | 0.0012 |
| 94: Right insula | 172: Left supplementary motor area | 10.4635 | 3.5393 | 0.0012 |
| 94: Right insula | 185: Left superior frontal gyrus, dorsolateral | 10.3268 | 3.8969 | 0.0008 |
| 95: Right inferior parietal gyrus, excluding supramarginal and angular gyri | 240: Left superior frontal gyrus, medial | 10.6299 | 4.1469 | 0.0008 |
| 105: Right supplementary motor area | 164: Left middle frontal gyrus | 8.5601 | 3.7318 | 0.0010 |

Table S7 The functional connectivities associated with subclinical depressive symptoms in remission of MDD (continued)

| Functional connectivities | | coefficient | t-value | *p*-value-FDR |
| --- | --- | --- | --- | --- |
| Region 1 | Region 2 |  |  |  |
| 105: Right supplementary motor area | 179: Left supramarginal gyrus | 10.0585 | 3.4421 | 0.0014 |
| 109: Right fusiform gyrus | 194: Left middle occipital gyrus | 10.2577 | 3.8271 | 0.0009 |
| 109: Right fusiform gyrus | 226: Left middle occipital gyrus | 11.2485 | 4.2300 | 0.0008 |
| 111: Right fusiform gyrus | 223: Left inferior occipital gyrus | 11.1297 | 3.6724 | 0.0010 |
| 111: Right fusiform gyrus | 243: Left calcarine fissure and surrounding cortex | 12.6480 | 3.5436 | 0.0012 |
| 112: Right fusiform gyrus | 190: Left lingual gyrus | 9.2764 | 3.4921 | 0.0013 |
| 114: Right precentral gyrus | 220: Left temporal pole: superior temporal gyrus | 10.3734 | 4.5101 | 0.0008 |
| 120: Right middle temporal gyrus | 129: Right parahippocampal gyrus | 11.8915 | 4.3005 | 0.0008 |
| 125: Right lenticular nucleus, putamen | 127: Right superior frontal gyrus, medial | 9.4331 | 4.1131 | 0.0008 |
| 125: Right lenticular nucleus, putamen | 158: Left precuneus | 9.8091 | 3.7249 | 0.0010 |
| 125: Right lenticular nucleus, putamen | 166: Left temporal pole: middle temporal gyrus | 7.9900 | 3.6088 | 0.0011 |
| 125: Right lenticular nucleus, putamen | 167: Left anterior cingulate & paracingulate gyri | 9.8763 | 4.1701 | 0.0008 |
| 125: Right lenticular nucleus, putamen | 175: Left hippocampus | 9.9823 | 3.4453 | 0.0014 |
| 125: Right lenticular nucleus, putamen | 187: Left lingual gyrus | 9.7580 | 3.7200 | 0.0010 |
| 129: Right parahippocampal gyrus | 140: Left postcentral gyrus | 12.4340 | 4.1905 | 0.0008 |
| 129: Right parahippocampal gyrus | 154: Left inferior parietal gyrus, excluding supramarginal and angular gyri | 11.5350 | 3.9107 | 0.0008 |
| 132: Right middle frontal gyrus | 140: Left postcentral gyrus | 11.8671 | 3.7893 | 0.0009 |
| 132: Right middle frontal gyrus | 178: Left angular gyrus | 10.3759 | 3.9072 | 0.0008 |
| 135: Left middle frontal gyrus | 176: Left angular gyrus | 9.0298 | 3.8538 | 0.0008 |
| 135: Left middle frontal gyrus | 233: Left middle frontal gyrus | 6.9856 | 3.6888 | 0.0010 |
| 135: Left middle frontal gyrus | 239: Left hippocampus | 11.6279 | 4.0652 | 0.0008 |
| 138: Left precuneus | 240: Left superior frontal gyrus, medial | 11.0564 | 3.9152 | 0.0008 |

Table S7 The functional connectivities associated with subclinical depressive symptoms in remission of MDD (continued)

| Functional connectivities | | coefficient | t-value | *p*-value-FDR |
| --- | --- | --- | --- | --- |
| Region 1 | Region 2 |  |  |  |
| 142: Left caudate nucleus | 185: Left superior frontal gyrus, dorsolateral | 9.2972 | 3.7026 | 0.0010 |
| 145: Left postcentral gyrus | 164: Left middle frontal gyrus | 12.1125 | 3.7217 | 0.0010 |
| 145: Left postcentral gyrus | 173: Left temporal pole: superior temporal gyrus | 10.2076 | 3.4468 | 0.0014 |
| 145: Left postcentral gyrus | 179: Left supramarginal gyrus | 13.4594 | 4.4726 | 0.0008 |
| 145: Left postcentral gyrus | 221: Left supramarginal gyrus | 10.4479 | 3.6603 | 0.0010 |
| 152: Left middle frontal gyrus | 189: Left insula | 11.8253 | 4.2426 | 0.0008 |
| 152: Left middle frontal gyrus | 222: Left insula | 9.0821 | 3.9672 | 0.0008 |
| 152: Left middle frontal gyrus | 250: Left postcentral gyrus | 9.6397 | 4.0707 | 0.0008 |
| 161: Left middle cingulate & paracingulate gyri | 172: Left supplementary motor area | 9.8498 | 3.5616 | 0.0012 |
| 167: Left anterior cingulate & paracingulate gyri | 178: Left angular gyrus | 10.4548 | 3.7145 | 0.0010 |
| 168: Left superior temporal gyrus | 222: Left insula | 9.4792 | 4.0002 | 0.0008 |
| 168: Left superior temporal gyrus | 242: Left insula | 10.4506 | 4.0919 | 0.0008 |
| 177: Right lateral orbital gyrus | 227: Left middle frontal gyrus | 9.9803 | 3.5227 | 0.0012 |
| 189: Left insula | 214: Left superior temporal gyrus | 10.7714 | 3.9168 | 0.0008 |
| 195: Left inferior frontal gyrus, triangular part | 229: Left postcentral gyrus | 10.7413 | 3.5778 | 0.0011 |
| 204: Left middle frontal gyrus | 232: Left middle cingulate & paracingulate gyri | 12.9531 | 4.5231 | 0.0008 |
| 208: Left middle cingulate & paracingulate gyri | 227: Left middle frontal gyrus | 9.2208 | 3.6295 | 0.0011 |
| 241: Left superior temporal gyrus | 242: Left insula | 12.2266 | 4.2301 | 0.0008 |

*p*-value-FDR: *p* value corrected by false discovery rate.

Table S8 The functional connectivities associated with somatic symptoms in remission of MDD

| Functional connectivities | | Coefficient | t-value | *p*-value-FDR |
| --- | --- | --- | --- | --- |
| Region 1 | Region 2 |  |  |  |
| 1: Right posterior orbitofrontal gyrus | 112: Right fusiform gyrus | 8.4721 | 3.8724 | 0.0011 |
| 1: Right posterior orbitofrontal gyrus | 139: Left inferior temporal gyrus | 6.8933 | 4.2989 | 0.0009 |
| 1: Right posterior orbitofrontal gyrus | 173: Left temporal pole: superior temporal gyrus | 8.9310 | 6.0774 | 0.0001 |
| 1: Right posterior orbitofrontal gyrus | 176: Left angular gyrus | 8.7762 | 4.4564 | 0.0008 |
| 1: Right posterior orbitofrontal gyrus | 178: Left angular gyrus | 7.2374 | 3.8828 | 0.0011 |
| 1: Right posterior orbitofrontal gyrus | 184: Left inferior temporal gyrus | 9.0954 | 5.5111 | 0.0001 |
| 1: Right posterior orbitofrontal gyrus | 198: Left superior temporal gyrus | 6.2198 | 4.1278 | 0.0010 |
| 1: Right posterior orbitofrontal gyrus | 217: Left supramarginal gyrus | 6.8100 | 3.8608 | 0.0011 |
| 1: Right posterior orbitofrontal gyrus | 221: Left supramarginal gyrus | 6.5528 | 4.2018 | 0.0010 |
| 1: Right posterior orbitofrontal gyrus | 247: Left middle occipital gyrus | 8.6775 | 4.3126 | 0.0009 |
| 2: Left posterior orbitofrontal gyrus | 80: Right hippocampus | 4.3880 | 3.2100 | 0.0027 |
| 3: Right anterior orbitofrontal gyrus | 36: Right angular gyrus | 7.1045 | 3.6063 | 0.0014 |
| 3: Right anterior orbitofrontal gyrus | 144: Left middle occipital gyrus | 7.4461 | 3.3492 | 0.0020 |
| 3: Right anterior orbitofrontal gyrus | 149: Left inferior parietal gyrus, excluding supramarginal and angular gyri | 6.1015 | 3.3744 | 0.0020 |
| 3: Right anterior orbitofrontal gyrus | 201: Left cuneus | 6.6731 | 3.5508 | 0.0015 |
| 3: Right anterior orbitofrontal gyrus | 243: Left calcarine fissure and surrounding cortex | 6.9072 | 3.3558 | 0.0020 |
| 7: Right gyrus rectus | 149: Left inferior parietal gyrus, excluding supramarginal and angular gyri | 6.5947 | 3.6170 | 0.0014 |
| 7: Right gyrus rectus | 216: Left superior parietal gyrus | 6.8644 | 3.4116 | 0.0018 |
| 8: Left gyrus rectus | 35: Right middle temporal gyrus | 6.2687 | 3.1791 | 0.0029 |
| 8: Left gyrus rectus | 149: Left inferior parietal gyrus, excluding supramarginal and angular gyri | 7.9079 | 4.3102 | 0.0009 |

Table S8 The functional connectivities associated with somatic symptoms in remission of MDD (continued)

| Functional connectivities | | Coefficient | t-value | *p*-value-FDR |
| --- | --- | --- | --- | --- |
| Region 1 | Region 2 |  |  |  |
| 8: Left gyrus rectus | 194: Left middle occipital gyrus | 8.4500 | 3.7015 | 0.0013 |
| 8: Left gyrus rectus | 216: Left superior parietal gyrus | 8.4809 | 3.8678 | 0.0011 |
| 8: Left gyrus rectus | 226: Left middle occipital gyrus | 6.9560 | 3.5142 | 0.0016 |
| 8: Left gyrus rectus | 247: Left middle occipital gyrus | 9.0624 | 3.3688 | 0.0020 |
| 9: Right olfactory cortex | 103: Right inferior occipital gyrus | 9.0348 | 4.0805 | 0.0010 |
| 9: Right olfactory cortex | 149: Left inferior parietal gyrus, excluding supramarginal and angular gyri | 7.7659 | 3.3356 | 0.0021 |
| 10: Left olfactory cortex | 149: Left inferior parietal gyrus, excluding supramarginal and angular gyri | 6.7778 | 3.3099 | 0.0022 |
| 12: Left lateral orbitofrontal gyrus | 81: Right temporal pole: superior temporal gyrus | 8.3763 | 3.3226 | 0.0021 |
| 12: Left lateral orbitofrontal gyrus | 179: Left supramarginal gyrus | 7.7754 | 3.2468 | 0.0025 |
| 13: Right inferior frontal gyrus, orbital part | 163: Left fusiform gyrus | 6.6480 | 3.7259 | 0.0013 |
| 13: Right inferior frontal gyrus, orbital part | 173: Left temporal pole: superior temporal gyrus | 6.9065 | 4.6927 | 0.0006 |
| 14: Left inferior frontal gyrus, orbital part | 44: Right postcentral gyrus | 6.2800 | 3.8088 | 0.0012 |
| 14: Left inferior frontal gyrus, orbital part | 114: Right precentral gyrus | 5.7912 | 4.0310 | 0.0011 |
| 15: Right precentral gyrus | 166: Left temporal pole: middle temporal gyrus | 6.3865 | 3.1849 | 0.0029 |
| 15: Right precentral gyrus | 173: Left temporal pole: superior temporal gyrus | 6.9116 | 4.5399 | 0.0007 |
| 15: Right precentral gyrus | 212: Left fusiform gyrus | 6.1624 | 3.8291 | 0.0012 |
| 15: Right precentral gyrus | 231: Left inferior occipital gyrus | 6.7113 | 3.7103 | 0.0013 |
| 16: Right superior frontal gyrus, dorsolateral | 70: Right middle frontal gyrus | 5.5324 | 3.5179 | 0.0016 |
| 16: Right superior frontal gyrus, dorsolateral | 162: Left precentral gyrus | 5.7440 | 3.7696 | 0.0012 |
| 16: Right superior frontal gyrus, dorsolateral | 180: Left precentral gyrus | 5.3257 | 3.8459 | 0.0011 |
| 16: Right superior frontal gyrus, dorsolateral | 195: Left inferior frontal gyrus, triangular part | 7.0880 | 3.6960 | 0.0013 |

Table S8 The functional connectivities associated with somatic symptoms in remission of MDD (continued)

| Functional connectivities | | Coefficient | t-value | *p*-value-FDR |
| --- | --- | --- | --- | --- |
| Region 1 | Region 2 |  |  |  |
| 16: Right superior frontal gyrus, dorsolateral | 204: Left middle frontal gyrus | 5.1696 | 3.7385 | 0.0012 |
| 16: Right superior frontal gyrus, dorsolateral | 250: Left postcentral gyrus | 5.4383 | 3.6259 | 0.0014 |
| 17: Right superior frontal gyrus, medial orbital (or ventromedial prefrontal cortex) | 115: Right lingual gyrus | 5.8618 | 3.4247 | 0.0018 |
| 18: Right postcentral gyrus | 139: Left inferior temporal gyrus | 5.7840 | 3.7298 | 0.0013 |
| 18: Right postcentral gyrus | 162: Left precentral gyrus | 6.7881 | 3.9288 | 0.0011 |
| 18: Right postcentral gyrus | 164: Left middle frontal gyrus | 6.7001 | 4.0353 | 0.0011 |
| 18: Right postcentral gyrus | 173: Left temporal pole: superior temporal gyrus | 6.4496 | 3.9238 | 0.0011 |
| 18: Right postcentral gyrus | 193: Left fusiform gyrus | 7.0525 | 3.8975 | 0.0011 |
| 18: Right postcentral gyrus | 195: Left inferior frontal gyrus, triangular part | 7.3101 | 4.3555 | 0.0009 |
| 18: Right postcentral gyrus | 227: Left middle frontal gyrus | 8.5677 | 4.6846 | 0.0006 |
| 19: Right supramarginal gyrus | 173: Left temporal pole: superior temporal gyrus | 5.3757 | 3.6362 | 0.0014 |
| 21: Right precentral gyrus | 172: Left supplementary motor area | 5.7189 | 3.5741 | 0.0014 |
| 21: Right precentral gyrus | 180: Left precentral gyrus | 5.1912 | 3.8490 | 0.0011 |
| 21: Right precentral gyrus | 227: Left middle frontal gyrus | 6.3775 | 3.9640 | 0.0011 |
| 23: Right middle frontal gyrus | 145: Left postcentral gyrus | 9.0861 | 4.1361 | 0.0010 |
| 23: Right middle frontal gyrus | 180: Left precentral gyrus | 5.6834 | 3.6103 | 0.0014 |
| 23: Right middle frontal gyrus | 195: Left inferior frontal gyrus, triangular part | 6.9478 | 3.6223 | 0.0014 |
| 23: Right middle frontal gyrus | 204: Left middle frontal gyrus | 6.4834 | 3.7183 | 0.0013 |
| 24: Right inferior temporal gyrus | 31: Right superior temporal gyrus | 4.8935 | 3.0969 | 0.0035 |
| 24: Right inferior temporal gyrus | 64: Right superior temporal gyrus | 6.7796 | 3.8775 | 0.0011 |
| 25: Right thalamus | 178: Left angular gyrus | 7.0205 | 3.3896 | 0.0019 |
| 28: Right heschl's gyrus | 163: Left fusiform gyrus | 7.2205 | 3.6454 | 0.0014 |

Table S8 The functional connectivities associated with somatic symptoms in remission of MDD (continued)

| Functional connectivities | | Coefficient | t-value | *p*-value-FDR |
| --- | --- | --- | --- | --- |
| Region 1 | Region 2 |  |  |  |
| 28: Right heschl's gyrus | 164: Left middle frontal gyrus | 7.1179 | 3.1910 | 0.0028 |
| 28: Right heschl's gyrus | 173: Left temporal pole: superior temporal gyrus | 5.2597 | 3.6450 | 0.0014 |
| 30: Right supramarginal gyrus | 173: Left temporal pole: superior temporal gyrus | 5.2027 | 3.5654 | 0.0014 |
| 31: Right superior temporal gyrus | 173: Left temporal pole: superior temporal gyrus | 5.4045 | 3.8639 | 0.0011 |
| 33: Right inferior frontal gyrus, triangular part | 139: Left inferior temporal gyrus | 7.4193 | 3.8717 | 0.0011 |
| 33: Right inferior frontal gyrus, triangular part | 173: Left temporal pole: superior temporal gyrus | 6.7850 | 3.8709 | 0.0011 |
| 34: Right precuneus | 52: Right insula | 7.6272 | 3.3695 | 0.0020 |
| 35: Right middle temporal gyrus | 244: Left anterior cingulate & paracingulate gyri | 8.1475 | 4.4659 | 0.0008 |
| 37: Right caudate nucleus | 88: Right fusiform gyrus | 7.1954 | 3.2232 | 0.0026 |
| 37: Right caudate nucleus | 103: Right inferior occipital gyrus | 8.1044 | 3.4192 | 0.0018 |
| 37: Right caudate nucleus | 163: Left fusiform gyrus | 8.3509 | 4.0899 | 0.0010 |
| 37: Right caudate nucleus | 175: Left hippocampus | 6.5878 | 3.6512 | 0.0014 |
| 37: Right caudate nucleus | 190: Left lingual gyrus | 6.4118 | 3.5882 | 0.0014 |
| 37: Right caudate nucleus | 198: Left superior temporal gyrus | 9.3753 | 3.9966 | 0.0011 |
| 38: Right temporal pole: middle temporal gyrus | 163: Left fusiform gyrus | 6.6597 | 4.1432 | 0.0010 |
| 38: Right temporal pole: middle temporal gyrus | 175: Left hippocampus | 6.4120 | 4.7807 | 0.0005 |
| 38: Right temporal pole: middle temporal gyrus | 217: Left supramarginal gyrus | 5.6663 | 3.2918 | 0.0023 |
| 38: Right temporal pole: middle temporal gyrus | 221: Left supramarginal gyrus | 5.7127 | 3.6376 | 0.0014 |
| 38: Right temporal pole: middle temporal gyrus | 222: Left insula | 5.8176 | 3.8622 | 0.0011 |
| 38: Right temporal pole: middle temporal gyrus | 239: Left hippocampus | 5.7261 | 4.0830 | 0.0010 |
| 39: Right middle cingulate & paracingulate gyri | 162: Left precentral gyrus | 5.5762 | 4.0044 | 0.0011 |
| 39: Right middle cingulate & paracingulate gyri | 163: Left fusiform gyrus | 10.8904 | 5.9321 | 0.0001 |
| 39: Right middle cingulate & paracingulate gyri | 173: Left temporal pole: superior temporal gyrus | 5.8955 | 3.7724 | 0.0012 |

Table S8 The functional connectivities associated with somatic symptoms in remission of MDD (continued)

| Functional connectivities | | Coefficient | t-value | *p*-value-FDR |
| --- | --- | --- | --- | --- |
| Region 1 | Region 2 |  |  |  |
| 39: Right middle cingulate & paracingulate gyri | 175: Left hippocampus | 6.1645 | 3.9182 | 0.0011 |
| 39: Right middle cingulate & paracingulate gyri | 180: Left precentral gyrus | 5.2281 | 4.0917 | 0.0010 |
| 39: Right middle cingulate & paracingulate gyri | 188: Left precentral gyrus | 6.7737 | 3.1948 | 0.0028 |
| 39: Right middle cingulate & paracingulate gyri | 195: Left inferior frontal gyrus, triangular part | 5.1938 | 3.4003 | 0.0018 |
| 39: Right middle cingulate & paracingulate gyri | 222: Left insula | 5.5039 | 3.6064 | 0.0014 |
| 39: Right middle cingulate & paracingulate gyri | 250: Left postcentral gyrus | 5.0514 | 3.7712 | 0.0012 |
| 40: Right postcentral gyrus | 70: Right middle frontal gyrus | 7.8389 | 3.7920 | 0.0012 |
| 40: Right postcentral gyrus | 102: Right superior frontal gyrus, dorsolateral | 5.9989 | 3.7045 | 0.0013 |
| 40: Right postcentral gyrus | 164: Left middle frontal gyrus | 7.0154 | 3.9279 | 0.0011 |
| 41: Right superior parietal gyrus | 178: Left angular gyrus | 7.8760 | 3.8918 | 0.0011 |
| 42: Right postcentral gyrus | 227: Left middle frontal gyrus | 5.8550 | 3.3304 | 0.0021 |
| 43: Right middle temporal gyrus | 203: Left superior frontal gyrus, medial | 4.9627 | 3.1546 | 0.0031 |
| 44: Right postcentral gyrus | 139: Left inferior temporal gyrus | 7.5595 | 4.0606 | 0.0010 |
| 44: Right postcentral gyrus | 164: Left middle frontal gyrus | 7.7679 | 4.2819 | 0.0009 |
| 44: Right postcentral gyrus | 166: Left temporal pole: middle temporal gyrus | 7.3843 | 3.8644 | 0.0011 |
| 44: Right postcentral gyrus | 173: Left temporal pole: superior temporal gyrus | 7.8379 | 5.7060 | 0.0001 |
| 44: Right postcentral gyrus | 193: Left fusiform gyrus | 8.7357 | 3.5295 | 0.0015 |
| 44: Right postcentral gyrus | 195: Left inferior frontal gyrus, triangular part | 6.6294 | 3.9939 | 0.0011 |
| 44: Right postcentral gyrus | 220: Left temporal pole: superior temporal gyrus | 6.2566 | 3.6377 | 0.0014 |
| 45: Right inferior temporal gyrus | 244: Left anterior cingulate & paracingulate gyri | 7.3461 | 4.0735 | 0.0010 |
| 46: Right cuneus | 203: Left superior frontal gyrus, medial | 6.6107 | 3.4972 | 0.0016 |
| 52: Right insula | 167: Left anterior cingulate & paracingulate gyri | 6.4507 | 3.5981 | 0.0014 |
| 52: Right insula | 173: Left temporal pole: superior temporal gyrus | 6.9295 | 3.8068 | 0.0012 |

Table S8 The functional connectivities associated with somatic symptoms in remission of MDD (continued)

| Functional connectivities | | Coefficient | t-value | *p*-value-FDR |
| --- | --- | --- | --- | --- |
| Region 1 | Region 2 |  |  |  |
| 52: Right insula | 178: Left angular gyrus | 8.7139 | 4.0922 | 0.0010 |
| 52: Right insula | 221: Left supramarginal gyrus | 6.8859 | 3.6424 | 0.0014 |
| 53: Right superior temporal gyrus | 173: Left temporal pole: superior temporal gyrus | 5.8849 | 4.1404 | 0.0010 |
| 54: Right precuneus | 62: Right anterior cingulate & paracingulate gyri | 5.4377 | 3.2828 | 0.0023 |
| 54: Right precuneus | 149: Left inferior parietal gyrus, excluding supramarginal and angular gyri | 6.4098 | 4.1532 | 0.0010 |
| 54: Right precuneus | 178: Left angular gyrus | 6.5413 | 4.0100 | 0.0011 |
| 54: Right precuneus | 181: Left inferior temporal gyrus | 5.5534 | 3.4916 | 0.0016 |
| 54: Right precuneus | 199: Left inferior parietal gyrus, excluding supramarginal and angular gyri | 7.1168 | 3.6036 | 0.0014 |
| 54: Right precuneus | 227: Left middle frontal gyrus | 5.6609 | 3.3717 | 0.0020 |
| 55: Right superior frontal gyrus, medial | 64: Right superior temporal gyrus | 6.1276 | 3.7883 | 0.0012 |
| 56: Right posterior cingulate cortex (retrosplenial) | 86: Right cuneus | 7.9965 | 3.3666 | 0.0020 |
| 56: Right posterior cingulate cortex (retrosplenial) | 101: Right inferior parietal gyrus, excluding supramarginal and angular gyri | 6.0269 | 3.4137 | 0.0018 |
| 56: Right posterior cingulate cortex (retrosplenial) | 243: Left calcarine fissure and surrounding cortex | 7.3139 | 3.4932 | 0.0016 |
| 58: Right postcentral gyrus | 173: Left temporal pole: superior temporal gyrus | 6.3296 | 3.8822 | 0.0011 |
| 59: Right middle temporal gyrus | 83: Right anterior cingulate & paracingulate gyri | 6.5415 | 3.4275 | 0.0018 |
| 59: Right middle temporal gyrus | 164: Left middle frontal gyrus | 6.3391 | 3.6122 | 0.0014 |
| 59: Right middle temporal gyrus | 173: Left temporal pole: superior temporal gyrus | 6.0235 | 3.5282 | 0.0015 |
| 59: Right middle temporal gyrus | 175: Left hippocampus | 6.6303 | 3.5778 | 0.0014 |
| 59: Right middle temporal gyrus | 226: Left middle occipital gyrus | 7.9466 | 3.9152 | 0.0011 |
| 60: Right caudate nucleus | 125: Right lenticular nucleus, putamen | 5.6718 | 3.4256 | 0.0018 |

Table S8 The functional connectivities associated with somatic symptoms in remission of MDD (continued)

| Functional connectivities | | Coefficient | t-value | *p*-value-FDR |
| --- | --- | --- | --- | --- |
| Region 1 | Region 2 |  |  |  |
| 62: Right anterior cingulate & paracingulate gyri | 88: Right fusiform gyrus | 7.6353 | 4.1917 | 0.0010 |
| 62: Right anterior cingulate & paracingulate gyri | 103: Right inferior occipital gyrus | 8.7110 | 3.8456 | 0.0011 |
| 62: Right anterior cingulate & paracingulate gyri | 124: Right calcarine fissure and surrounding cortex | 6.4251 | 3.0887 | 0.0036 |
| 62: Right anterior cingulate & paracingulate gyri | 125: Right lenticular nucleus, putamen | 5.1596 | 3.6195 | 0.0014 |
| 62: Right anterior cingulate & paracingulate gyri | 149: Left inferior parietal gyrus, excluding supramarginal and angular gyri | 6.2454 | 3.4673 | 0.0017 |
| 62: Right anterior cingulate & paracingulate gyri | 180: Left precentral gyrus | 7.1969 | 4.9357 | 0.0005 |
| 62: Right anterior cingulate & paracingulate gyri | 204: Left middle frontal gyrus | 5.8748 | 3.7494 | 0.0012 |
| 63: Right middle cingulate & paracingulate gyri | 139: Left inferior temporal gyrus | 7.1240 | 3.5655 | 0.0014 |
| 63: Right middle cingulate & paracingulate gyri | 164: Left middle frontal gyrus | 7.7891 | 4.3890 | 0.0008 |
| 63: Right middle cingulate & paracingulate gyri | 173: Left temporal pole: superior temporal gyrus | 6.0779 | 4.2810 | 0.0009 |
| 63: Right middle cingulate & paracingulate gyri | 180: Left precentral gyrus | 4.8256 | 3.4846 | 0.0016 |
| 63: Right middle cingulate & paracingulate gyri | 195: Left inferior frontal gyrus, triangular part | 6.0598 | 3.4999 | 0.0016 |
| 64: Right superior temporal gyrus | 133: Right middle cingulate & paracingulate gyri | 5.7558 | 3.1851 | 0.0029 |
| 64: Right superior temporal gyrus | 141: Left lenticular nucleus, putamen | 7.8528 | 3.2901 | 0.0023 |
| 64: Right superior temporal gyrus | 173: Left temporal pole: superior temporal gyrus | 4.9417 | 3.5968 | 0.0014 |
| 65: Right postcentral gyrus | 93: Right middle cingulate & paracingulate gyri | 6.9993 | 3.4807 | 0.0016 |
| 67: Right supplementary motor area | 77: Right middle frontal gyrus | 5.7094 | 3.4486 | 0.0017 |
| 67: Right supplementary motor area | 173: Left temporal pole: superior temporal gyrus | 6.8454 | 3.5635 | 0.0014 |
| 67: Right supplementary motor area | 180: Left precentral gyrus | 6.0546 | 4.0376 | 0.0011 |
| 69: Right middle temporal gyrus | 139: Left inferior temporal gyrus | 5.5878 | 3.5493 | 0.0015 |
| 69: Right middle temporal gyrus | 164: Left middle frontal gyrus | 6.9017 | 3.4436 | 0.0017 |
| 69: Right middle temporal gyrus | 173: Left temporal pole: superior temporal gyrus | 6.7869 | 3.8917 | 0.0011 |

Table S8 The functional connectivities associated with somatic symptoms in remission of MDD (continued)

| Functional connectivities | | Coefficient | t-value | *p*-value-FDR |
| --- | --- | --- | --- | --- |
| Region 1 | Region 2 |  |  |  |
| 69: Right middle temporal gyrus | 178: Left angular gyrus | 8.4516 | 3.8449 | 0.0011 |
| 70: Right middle frontal gyrus | 74: Right anterior cingulate & paracingulate gyri | 4.9036 | 3.1962 | 0.0028 |
| 70: Right middle frontal gyrus | 75: Right temporal pole: middle temporal gyrus | 5.2119 | 3.3177 | 0.0021 |
| 70: Right middle frontal gyrus | 102: Right superior frontal gyrus, dorsolateral | 5.9497 | 4.1658 | 0.0010 |
| 70: Right middle frontal gyrus | 133: Right middle cingulate & paracingulate gyri | 5.9265 | 3.5813 | 0.0014 |
| 70: Right middle frontal gyrus | 135: Left middle frontal gyrus | 6.4156 | 3.7178 | 0.0013 |
| 70: Right middle frontal gyrus | 206: Left superior frontal gyrus, dorsolateral | 6.3099 | 4.0022 | 0.0011 |
| 70: Right middle frontal gyrus | 222: Left insula | 7.4701 | 3.5017 | 0.0016 |
| 70: Right middle frontal gyrus | 227: Left middle frontal gyrus | 5.6796 | 3.7429 | 0.0012 |
| 70: Right middle frontal gyrus | 245: Left caudate nucleus | 7.0653 | 3.3769 | 0.0019 |
| 71: Right superior frontal gyrus, dorsolateral | 162: Left precentral gyrus | 5.0419 | 3.6786 | 0.0013 |
| 71: Right superior frontal gyrus, dorsolateral | 180: Left precentral gyrus | 5.7415 | 4.4501 | 0.0008 |
| 71: Right superior frontal gyrus, dorsolateral | 250: Left postcentral gyrus | 5.4702 | 3.9205 | 0.0011 |
| 72: Right superior frontal gyrus, medial orbital (or ventromedial prefrontal cortex) | 149: Left inferior parietal gyrus, excluding supramarginal and angular gyri | 6.7861 | 3.7936 | 0.0012 |
| 72: Right superior frontal gyrus, medial orbital (or ventromedial prefrontal cortex) | 212: Left fusiform gyrus | 7.6262 | 3.6743 | 0.0013 |
| 72: Right superior frontal gyrus, medial orbital (or ventromedial prefrontal cortex) | 216: Left superior parietal gyrus | 6.6990 | 3.4072 | 0.0018 |
| 73: Right middle frontal gyrus | 173: Left temporal pole: superior temporal gyrus | 6.3819 | 3.9835 | 0.0011 |
| 73: Right middle frontal gyrus | 178: Left angular gyrus | 4.9265 | 3.3572 | 0.0020 |
| 74: Right anterior cingulate & paracingulate gyri | 88: Right fusiform gyrus | 6.0452 | 3.4526 | 0.0017 |
| 74: Right anterior cingulate & paracingulate gyri | 140: Left postcentral gyrus | 6.9618 | 3.6212 | 0.0014 |

Table S8 The functional connectivities associated with somatic symptoms in remission of MDD (continued)

| Functional connectivities | | Coefficient | t-value | *p*-value-FDR |
| --- | --- | --- | --- | --- |
| Region 1 | Region 2 |  |  |  |
| 74: Right anterior cingulate & paracingulate gyri | 169: Left calcarine fissure and surrounding cortex | 6.6400 | 3.5038 | 0.0016 |
| 74: Right anterior cingulate & paracingulate gyri | 180: Left precentral gyrus | 6.5723 | 3.9709 | 0.0011 |
| 75: Right temporal pole: middle temporal gyrus | 171: Left precuneus | 5.0660 | 3.3289 | 0.0021 |
| 75: Right temporal pole: middle temporal gyrus | 175: Left hippocampus | 5.7311 | 4.0076 | 0.0011 |
| 76: Right middle cingulate & paracingulate gyri | 173: Left temporal pole: superior temporal gyrus | 6.1397 | 3.7284 | 0.0013 |
| 77: Right middle frontal gyrus | 159: Left supplementary motor area | 6.1921 | 3.6610 | 0.0014 |
| 77: Right middle frontal gyrus | 173: Left temporal pole: superior temporal gyrus | 7.7072 | 3.6231 | 0.0014 |
| 77: Right middle frontal gyrus | 180: Left precentral gyrus | 6.3329 | 3.8858 | 0.0011 |
| 77: Right middle frontal gyrus | 204: Left middle frontal gyrus | 5.9801 | 3.1928 | 0.0028 |
| 78: Right fusiform gyrus | 195: Left inferior frontal gyrus, triangular part | 6.1113 | 3.3674 | 0.0020 |
| 79: Right superior temporal gyrus | 173: Left temporal pole: superior temporal gyrus | 6.1964 | 3.6410 | 0.0014 |
| 80: Right hippocampus | 173: Left temporal pole: superior temporal gyrus | 5.5309 | 3.5217 | 0.0015 |
| 81: Right temporal pole: superior temporal gyrus | 163: Left fusiform gyrus | 7.1986 | 3.7114 | 0.0013 |
| 81: Right temporal pole: superior temporal gyrus | 173: Left temporal pole: superior temporal gyrus | 6.4352 | 3.8920 | 0.0011 |
| 81: Right temporal pole: superior temporal gyrus | 175: Left hippocampus | 6.5212 | 3.7874 | 0.0012 |
| 81: Right temporal pole: superior temporal gyrus | 207: Left middle temporal gyrus | 5.8863 | 3.4448 | 0.0017 |
| 81: Right temporal pole: superior temporal gyrus | 212: Left fusiform gyrus | 5.8189 | 3.4222 | 0.0018 |
| 81: Right temporal pole: superior temporal gyrus | 217: Left supramarginal gyrus | 5.9138 | 3.9396 | 0.0011 |
| 82: Right superior parietal gyrus | 181: Left inferior temporal gyrus | 6.8781 | 3.7806 | 0.0012 |
| 84: Right insula | 173: Left temporal pole: superior temporal gyrus | 6.1656 | 4.2465 | 0.0010 |
| 84: Right insula | 212: Left fusiform gyrus | 6.7448 | 3.8869 | 0.0011 |
| 84: Right insula | 215: Left inferior temporal gyrus | 7.5861 | 3.4753 | 0.0016 |
| 86: Right cuneus | 234: Left thalamus | 7.3711 | 3.2454 | 0.0025 |

Table S8 The functional connectivities associated with somatic symptoms in remission of MDD (continued)

| Functional connectivities | | Coefficient | t-value | *p*-value-FDR |
| --- | --- | --- | --- | --- |
| Region 1 | Region 2 |  |  |  |
| 87: Right superior frontal gyrus, dorsolateral | 160: Left middle cingulate & paracingulate gyri | 4.5138 | 3.4328 | 0.0018 |
| 87: Right superior frontal gyrus, dorsolateral | 172: Left supplementary motor area | 5.9855 | 3.6631 | 0.0014 |
| 87: Right superior frontal gyrus, dorsolateral | 227: Left middle frontal gyrus | 5.3267 | 3.4294 | 0.0018 |
| 88: Right fusiform gyrus | 139: Left inferior temporal gyrus | 5.3783 | 3.3375 | 0.0021 |
| 88: Right fusiform gyrus | 173: Left temporal pole: superior temporal gyrus | 6.1351 | 3.8528 | 0.0011 |
| 88: Right fusiform gyrus | 177: Right lateral orbital gyrus | 6.8076 | 3.7924 | 0.0012 |
| 89: Right middle frontal gyrus | 103: Right inferior occipital gyrus | 8.6254 | 3.8230 | 0.0012 |
| 89: Right middle frontal gyrus | 180: Left precentral gyrus | 6.3397 | 4.1486 | 0.0010 |
| 89: Right middle frontal gyrus | 185: Left superior frontal gyrus, dorsolateral | 5.4489 | 3.6334 | 0.0014 |
| 89: Right middle frontal gyrus | 218: Left precentral gyrus | 6.6789 | 3.8204 | 0.0012 |
| 89: Right middle frontal gyrus | 227: Left middle frontal gyrus | 5.3291 | 3.6800 | 0.0013 |
| 91: Right inferior temporal gyrus | 92: Right insula | 6.2443 | 3.0896 | 0.0036 |
| 91: Right inferior temporal gyrus | 141: Left lenticular nucleus, putamen | 7.0570 | 3.3410 | 0.0021 |
| 91: Right inferior temporal gyrus | 174: Left cuneus | 7.0731 | 3.5936 | 0.0014 |
| 91: Right inferior temporal gyrus | 178: Left angular gyrus | 7.7606 | 4.6175 | 0.0007 |
| 91: Right inferior temporal gyrus | 194: Left middle occipital gyrus | 6.4344 | 4.1560 | 0.0010 |
| 91: Right inferior temporal gyrus | 216: Left superior parietal gyrus | 6.6220 | 4.2409 | 0.0010 |
| 91: Right inferior temporal gyrus | 221: Left supramarginal gyrus | 5.1069 | 3.4761 | 0.0016 |
| 91: Right inferior temporal gyrus | 226: Left middle occipital gyrus | 7.2242 | 4.0829 | 0.0010 |
| 92: Right insula | 173: Left temporal pole: superior temporal gyrus | 6.4923 | 4.0166 | 0.0011 |
| 92: Right insula | 181: Left inferior temporal gyrus | 6.6760 | 3.1469 | 0.0031 |
| 94: Right insula | 163: Left fusiform gyrus | 8.7847 | 4.5900 | 0.0007 |
| 94: Right insula | 173: Left temporal pole: superior temporal gyrus | 6.6207 | 4.7660 | 0.0005 |

Table S8 The functional connectivities associated with somatic symptoms in remission of MDD (continued)

| Functional connectivities | | Coefficient | t-value | *p*-value-FDR |
| --- | --- | --- | --- | --- |
| Region 1 | Region 2 |  |  |  |
| 94: Right insula | 180: Left precentral gyrus | 6.0064 | 3.8634 | 0.0011 |
| 94: Right insula | 212: Left fusiform gyrus | 6.2312 | 3.7150 | 0.0013 |
| 95: Right inferior parietal gyrus, excluding supramarginal and angular gyri | 131: Right lingual gyrus | 5.4705 | 3.2748 | 0.0023 |
| 96: Right middle temporal gyrus | 164: Left middle frontal gyrus | 5.9773 | 3.2613 | 0.0024 |
| 96: Right middle temporal gyrus | 201: Left cuneus | 5.3475 | 3.3740 | 0.0020 |
| 98: Right fusiform gyrus | 210: Left hippocampus | 5.4786 | 3.1732 | 0.0029 |
| 99: Right lenticular nucleus, putamen | 162: Left precentral gyrus | 8.7104 | 4.9420 | 0.0005 |
| 99: Right lenticular nucleus, putamen | 163: Left fusiform gyrus | 7.4562 | 3.4290 | 0.0018 |
| 99: Right lenticular nucleus, putamen | 173: Left temporal pole: superior temporal gyrus | 8.7616 | 4.5854 | 0.0007 |
| 99: Right lenticular nucleus, putamen | 212: Left fusiform gyrus | 6.6975 | 3.6466 | 0.0014 |
| 100: Right insula | 173: Left temporal pole: superior temporal gyrus | 5.6863 | 3.8249 | 0.0012 |
| 100: Right insula | 212: Left fusiform gyrus | 5.4209 | 3.2015 | 0.0028 |
| 101: Right inferior parietal gyrus, excluding supramarginal and angular gyri | 173: Left temporal pole: superior temporal gyrus | 5.4531 | 3.7828 | 0.0012 |
| 102: Right superior frontal gyrus, dorsolateral | 103: Right inferior occipital gyrus | 7.7814 | 3.7403 | 0.0012 |
| 102: Right superior frontal gyrus, dorsolateral | 105: Right supplementary motor area | 5.9842 | 3.2544 | 0.0024 |
| 102: Right superior frontal gyrus, dorsolateral | 168: Left superior temporal gyrus | 6.8344 | 3.2757 | 0.0023 |
| 102: Right superior frontal gyrus, dorsolateral | 169: Left calcarine fissure and surrounding cortex | 8.5731 | 4.0614 | 0.0010 |
| 102: Right superior frontal gyrus, dorsolateral | 180: Left precentral gyrus | 5.9101 | 3.6541 | 0.0014 |
| 102: Right superior frontal gyrus, dorsolateral | 185: Left superior frontal gyrus, dorsolateral | 5.4195 | 3.8182 | 0.0012 |
| 102: Right superior frontal gyrus, dorsolateral | 222: Left insula | 5.6036 | 3.5391 | 0.0015 |
| 103: Right inferior occipital gyrus | 172: Left supplementary motor area | 7.9343 | 3.7673 | 0.0012 |

Table S8 The functional connectivities associated with somatic symptoms in remission of MDD (continued)

| Functional connectivities | | Coefficient | t-value | *p*-value-FDR |
| --- | --- | --- | --- | --- |
| Region 1 | Region 2 |  |  |  |
| 103: Right inferior occipital gyrus | 244: Left anterior cingulate & paracingulate gyri | 6.8349 | 3.6233 | 0.0014 |
| 104: Right inferior frontal gyrus, triangular part | 173: Left temporal pole: superior temporal gyrus | 7.4181 | 3.4492 | 0.0017 |
| 105: Right supplementary motor area | 162: Left precentral gyrus | 7.2463 | 4.1973 | 0.0010 |
| 105: Right supplementary motor area | 173: Left temporal pole: superior temporal gyrus | 5.3311 | 3.4297 | 0.0018 |
| 105: Right supplementary motor area | 180: Left precentral gyrus | 6.1926 | 4.1752 | 0.0010 |
| 105: Right supplementary motor area | 186: Left posterior cingulate gyrus | 9.2107 | 4.2868 | 0.0009 |
| 107: Right inferior frontal gyrus, triangular part | 135: Left middle frontal gyrus | 6.5913 | 3.2578 | 0.0024 |
| 107: Right inferior frontal gyrus, triangular part | 139: Left inferior temporal gyrus | 6.4045 | 3.6094 | 0.0014 |
| 107: Right inferior frontal gyrus, triangular part | 173: Left temporal pole: superior temporal gyrus | 6.5189 | 3.5935 | 0.0014 |
| 107: Right inferior frontal gyrus, triangular part | 227: Left middle frontal gyrus | 7.2094 | 4.1754 | 0.0010 |
| 107: Right inferior frontal gyrus, triangular part | 231: Left inferior occipital gyrus | 6.7495 | 3.5505 | 0.0015 |
| 109: Right fusiform gyrus | 178: Left angular gyrus | 8.1221 | 3.6852 | 0.0013 |
| 109: Right fusiform gyrus | 194: Left middle occipital gyrus | 6.3040 | 3.4820 | 0.0016 |
| 109: Right fusiform gyrus | 216: Left superior parietal gyrus | 6.4107 | 3.3536 | 0.0020 |
| 110: Right inferior frontal gyrus, opercular part | 173: Left temporal pole: superior temporal gyrus | 7.2452 | 4.3846 | 0.0008 |
| 111: Right fusiform gyrus | 171: Left precuneus | 5.8564 | 3.7029 | 0.0013 |
| 112: Right fusiform gyrus | 129: Right parahippocampal gyrus | 7.6463 | 3.8448 | 0.0011 |
| 114: Right precentral gyrus | 157: Left insula | 6.2755 | 3.6313 | 0.0014 |
| 114: Right precentral gyrus | 164: Left middle frontal gyrus | 6.4667 | 3.5349 | 0.0015 |
| 114: Right precentral gyrus | 166: Left temporal pole: middle temporal gyrus | 5.9174 | 4.4028 | 0.0008 |
| 114: Right precentral gyrus | 173: Left temporal pole: superior temporal gyrus | 6.1116 | 4.1710 | 0.0010 |
| 114: Right precentral gyrus | 181: Left inferior temporal gyrus | 6.9192 | 4.3061 | 0.0009 |
| 114: Right precentral gyrus | 203: Left superior frontal gyrus, medial | 4.7261 | 3.4198 | 0.0018 |

Table S8 The functional connectivities associated with somatic symptoms in remission of MDD (continued)

| Functional connectivities | | Coefficient | t-value | *p*-value-FDR |
| --- | --- | --- | --- | --- |
| Region 1 | Region 2 |  |  |  |
| 114: Right precentral gyrus | 220: Left temporal pole: superior temporal gyrus | 6.4122 | 4.1010 | 0.0010 |
| 114: Right precentral gyrus | 236: Left caudate nucleus | 6.1430 | 3.3610 | 0.0020 |
| 115: Right lingual gyrus | 173: Left temporal pole: superior temporal gyrus | 6.6008 | 3.3287 | 0.0021 |
| 116: Right thalamus | 184: Left inferior temporal gyrus | 4.9626 | 3.1921 | 0.0028 |
| 116: Right thalamus | 243: Left calcarine fissure and surrounding cortex | 7.3169 | 3.4004 | 0.0018 |
| 117: Right superior frontal gyrus, medial orbital (or ventromedial prefrontal cortex) | 175: Left hippocampus | 6.8316 | 3.7048 | 0.0013 |
| 117: Right superior frontal gyrus, medial orbital (or ventromedial prefrontal cortex) | 212: Left fusiform gyrus | 7.4292 | 3.5564 | 0.0015 |
| 120: Right middle temporal gyrus | 133: Right middle cingulate & paracingulate gyri | 6.0800 | 3.2200 | 0.0027 |
| 120: Right middle temporal gyrus | 160: Left middle cingulate & paracingulate gyri | 5.7212 | 3.5854 | 0.0014 |
| 120: Right middle temporal gyrus | 164: Left middle frontal gyrus | 7.3335 | 3.5766 | 0.0014 |
| 120: Right middle temporal gyrus | 173: Left temporal pole: superior temporal gyrus | 5.9759 | 3.5947 | 0.0014 |
| 120: Right middle temporal gyrus | 209: Left superior parietal gyrus | 6.9843 | 3.4968 | 0.0016 |
| 120: Right middle temporal gyrus | 226: Left middle occipital gyrus | 7.1584 | 3.6857 | 0.0013 |
| 121: Right temporal pole: middle temporal gyrus | 163: Left fusiform gyrus | 6.0207 | 3.7766 | 0.0012 |
| 121: Right temporal pole: middle temporal gyrus | 175: Left hippocampus | 6.6591 | 5.2436 | 0.0003 |
| 121: Right temporal pole: middle temporal gyrus | 179: Left supramarginal gyrus | 5.2781 | 3.3253 | 0.0021 |
| 121: Right temporal pole: middle temporal gyrus | 217: Left supramarginal gyrus | 6.0198 | 4.1856 | 0.0010 |
| 121: Right temporal pole: middle temporal gyrus | 221: Left supramarginal gyrus | 4.7293 | 3.4820 | 0.0016 |
| 121: Right temporal pole: middle temporal gyrus | 222: Left insula | 4.6492 | 3.4153 | 0.0018 |
| 122: Right inferior frontal gyrus, opercular part | 135: Left middle frontal gyrus | 5.9470 | 3.3344 | 0.0021 |
| 122: Right inferior frontal gyrus, opercular part | 173: Left temporal pole: superior temporal gyrus | 6.7679 | 3.4911 | 0.0016 |

Table S8 The functional connectivities associated with somatic symptoms in remission of MDD (continued)

| Functional connectivities | | Coefficient | t-value | *p*-value-FDR |
| --- | --- | --- | --- | --- |
| Region 1 | Region 2 |  |  |  |
| 122: Right inferior frontal gyrus, opercular part | 185: Left superior frontal gyrus, dorsolateral | 6.3754 | 4.3656 | 0.0009 |
| 122: Right inferior frontal gyrus, opercular part | 227: Left middle frontal gyrus | 4.6073 | 3.2765 | 0.0023 |
| 122: Right inferior frontal gyrus, opercular part | 230: Left precuneus | 6.6872 | 3.1028 | 0.0035 |
| 122: Right inferior frontal gyrus, opercular part | 241: Left superior temporal gyrus | 5.9604 | 3.1689 | 0.0030 |
| 124: Right calcarine fissure and surrounding cortex | 173: Left temporal pole: superior temporal gyrus | 5.7672 | 3.5629 | 0.0014 |
| 125: Right lenticular nucleus, putamen | 140: Left postcentral gyrus | 6.3430 | 3.3231 | 0.0021 |
| 125: Right lenticular nucleus, putamen | 158: Left precuneus | 6.3616 | 3.6392 | 0.0014 |
| 125: Right lenticular nucleus, putamen | 162: Left precentral gyrus | 6.4435 | 3.7652 | 0.0012 |
| 125: Right lenticular nucleus, putamen | 168: Left superior temporal gyrus | 6.5272 | 3.8866 | 0.0011 |
| 125: Right lenticular nucleus, putamen | 178: Left angular gyrus | 8.4039 | 3.8477 | 0.0011 |
| 125: Right lenticular nucleus, putamen | 187: Left lingual gyrus | 6.2629 | 3.5850 | 0.0014 |
| 125: Right lenticular nucleus, putamen | 212: Left fusiform gyrus | 7.2420 | 3.8112 | 0.0012 |
| 125: Right lenticular nucleus, putamen | 227: Left middle frontal gyrus | 5.7606 | 3.5755 | 0.0014 |
| 126: Right middle frontal gyrus | 173: Left temporal pole: superior temporal gyrus | 6.7760 | 4.0997 | 0.0010 |
| 128: Right cuneus | 184: Left inferior temporal gyrus | 6.8899 | 3.8414 | 0.0011 |
| 129: Right parahippocampal gyrus | 163: Left fusiform gyrus | 10.1855 | 6.5523 | 0.0000 |
| 129: Right parahippocampal gyrus | 168: Left superior temporal gyrus | 6.8028 | 4.2700 | 0.0009 |
| 129: Right parahippocampal gyrus | 173: Left temporal pole: superior temporal gyrus | 7.2390 | 4.7851 | 0.0005 |
| 129: Right parahippocampal gyrus | 175: Left hippocampus | 7.5821 | 5.7529 | 0.0001 |
| 129: Right parahippocampal gyrus | 179: Left supramarginal gyrus | 7.2121 | 3.9266 | 0.0011 |
| 129: Right parahippocampal gyrus | 180: Left precentral gyrus | 7.3545 | 3.7752 | 0.0012 |
| 129: Right parahippocampal gyrus | 187: Left lingual gyrus | 7.6662 | 4.8028 | 0.0005 |
| 129: Right parahippocampal gyrus | 190: Left lingual gyrus | 6.3381 | 4.1909 | 0.0010 |

Table S8 The functional connectivities associated with somatic symptoms in remission of MDD (continued)

| Functional connectivities | | Coefficient | t-value | *p*-value-FDR |
| --- | --- | --- | --- | --- |
| Region 1 | Region 2 |  |  |  |
| 129: Right parahippocampal gyrus | 212: Left fusiform gyrus | 7.1203 | 4.7614 | 0.0005 |
| 129: Right parahippocampal gyrus | 217: Left supramarginal gyrus | 7.4390 | 4.4733 | 0.0008 |
| 129: Right parahippocampal gyrus | 221: Left supramarginal gyrus | 7.3420 | 4.5673 | 0.0007 |
| 129: Right parahippocampal gyrus | 229: Left postcentral gyrus | 5.5209 | 3.2933 | 0.0023 |
| 131: Right lingual gyrus | 243: Left calcarine fissure and surrounding cortex | 7.4392 | 4.1248 | 0.0010 |
| 132: Right middle frontal gyrus | 178: Left angular gyrus | 5.9661 | 3.2609 | 0.0024 |
| 132: Right middle frontal gyrus | 180: Left precentral gyrus | 6.3901 | 3.7799 | 0.0012 |
| 133: Right middle cingulate & paracingulate gyri | 159: Left supplementary motor area | 4.9961 | 3.5294 | 0.0015 |
| 133: Right middle cingulate & paracingulate gyri | 180: Left precentral gyrus | 6.2705 | 4.8113 | 0.0005 |
| 133: Right middle cingulate & paracingulate gyri | 185: Left superior frontal gyrus, dorsolateral | 4.8111 | 3.7606 | 0.0012 |
| 133: Right middle cingulate & paracingulate gyri | 204: Left middle frontal gyrus | 5.1611 | 3.4256 | 0.0018 |
| 134: Right precentral gyrus | 234: Left thalamus | 8.8716 | 3.9093 | 0.0011 |
| 135: Left middle frontal gyrus | 149: Left inferior parietal gyrus, excluding supramarginal and angular gyri | 5.9900 | 3.3505 | 0.0020 |
| 135: Left middle frontal gyrus | 189: Left insula | 10.1301 | 4.4225 | 0.0008 |
| 135: Left middle frontal gyrus | 199: Left inferior parietal gyrus, excluding supramarginal and angular gyri | 5.5708 | 3.4909 | 0.0016 |
| 138: Left precuneus | 151: Left insula | 8.7990 | 3.5657 | 0.0014 |
| 139: Left inferior temporal gyrus | 210: Left hippocampus | 8.1864 | 3.3641 | 0.0020 |
| 142: Left caudate nucleus | 212: Left fusiform gyrus | 10.7822 | 4.3256 | 0.0009 |
| 146: Left thalamus | 163: Left fusiform gyrus | 6.6417 | 3.1531 | 0.0031 |
| 149: Left inferior parietal gyrus, excluding supramarginal and angular gyri | 203: Left superior frontal gyrus, medial | 5.7078 | 3.4499 | 0.0017 |

Table S8 The functional connectivities associated with somatic symptoms in remission of MDD (continued)

| Functional connectivities | | Coefficient | t-value | *p*-value-FDR |
| --- | --- | --- | --- | --- |
| Region 1 | Region 2 |  |  |  |
| 149: Left inferior parietal gyrus, excluding supramarginal and angular gyri | 244: Left anterior cingulate & paracingulate gyri | 9.0676 | 4.5765 | 0.0007 |
| 150: Left anterior cingulate & paracingulate  gyri | 163: Left fusiform gyrus | 7.1531 | 3.4509 | 0.0017 |
| 150: Left anterior cingulate & paracingulate  gyri | 180: Left precentral gyrus | 6.7797 | 3.6255 | 0.0014 |
| 150: Left anterior cingulate & paracingulate  gyri | 222: Left insula | 7.6369 | 3.6299 | 0.0014 |
| 152: Left middle frontal gyrus | 159: Left supplementary motor area | 5.8049 | 3.4688 | 0.0017 |
| 152: Left middle frontal gyrus | 162: Left precentral gyrus | 5.0468 | 4.0982 | 0.0010 |
| 152: Left middle frontal gyrus | 180: Left precentral gyrus | 7.2393 | 5.1975 | 0.0003 |
| 152: Left middle frontal gyrus | 250: Left postcentral gyrus | 6.2214 | 3.9460 | 0.0011 |
| 159: Left supplementary motor area | 224: Left superior frontal gyrus, medial | 4.6311 | 3.4235 | 0.0018 |
| 160: Left middle cingulate & paracingulate gyri | 162: Left precentral gyrus | 5.1304 | 3.6969 | 0.0013 |
| 160: Left middle cingulate & paracingulate gyri | 163: Left fusiform gyrus | 7.9642 | 3.8506 | 0.0011 |
| 160: Left middle cingulate & paracingulate gyri | 173: Left temporal pole: superior temporal gyrus | 5.8348 | 3.8046 | 0.0012 |
| 160: Left middle cingulate & paracingulate gyri | 175: Left hippocampus | 5.8310 | 3.4821 | 0.0016 |
| 160: Left middle cingulate & paracingulate gyri | 180: Left precentral gyrus | 5.6704 | 3.6300 | 0.0014 |
| 160: Left middle cingulate & paracingulate gyri | 234: Left thalamus | 6.9486 | 3.5985 | 0.0014 |
| 162: Left precentral gyrus | 180: Left precentral gyrus | 6.9281 | 4.1905 | 0.0010 |
| 162: Left precentral gyrus | 193: Left fusiform gyrus | 5.6850 | 3.4922 | 0.0016 |
| 162: Left precentral gyrus | 224: Left superior frontal gyrus, medial | 5.6617 | 3.5799 | 0.0014 |
| 162: Left precentral gyrus | 229: Left postcentral gyrus | 5.7758 | 3.6084 | 0.0014 |

Table S8 The functional connectivities associated with somatic symptoms in remission of MDD (continued)

| Functional connectivities | | Coefficient | t-value | *p*-value-FDR |
| --- | --- | --- | --- | --- |
| Region 1 | Region 2 |  |  |  |
| 162: Left precentral gyrus | 234: Left thalamus | 7.2059 | 3.3428 | 0.0020 |
| 162: Left precentral gyrus | 249: Left supplementary motor area | 6.9692 | 4.0840 | 0.0010 |
| 162: Left precentral gyrus | 250: Left postcentral gyrus | 5.4447 | 3.6997 | 0.0013 |
| 164: Left middle frontal gyrus | 176: Left angular gyrus | 7.2695 | 3.0860 | 0.0036 |
| 165: Left anterior cingulate & paracingulate gyri | 180: Left precentral gyrus | 5.7859 | 3.5363 | 0.0015 |
| 167: Left anterior cingulate & paracingulate gyri | 222: Left insula | 5.9052 | 3.4797 | 0.0016 |
| 168: Left superior temporal gyrus | 195: Left inferior frontal gyrus, triangular part | 7.3825 | 4.5117 | 0.0008 |
| 169: Left calcarine fissure and surrounding cortex | 172: Left supplementary motor area | 7.8552 | 3.7117 | 0.0013 |
| 173: Left temporal pole: superior temporal gyrus | 210: Left hippocampus | 8.7319 | 3.8130 | 0.0012 |
| 173: Left temporal pole: superior temporal gyrus | 214: Left superior temporal gyrus | 6.3585 | 3.8499 | 0.0011 |
| 179: Left supramarginal gyrus | 180: Left precentral gyrus | 6.4855 | 3.6206 | 0.0014 |
| 180: Left precentral gyrus | 208: Left middle cingulate & paracingulate gyri | 5.6052 | 4.0723 | 0.0010 |
| 180: Left precentral gyrus | 222: Left insula | 6.1687 | 4.0008 | 0.0011 |
| 180: Left precentral gyrus | 224: Left superior frontal gyrus, medial | 5.6747 | 3.7981 | 0.0012 |
| 180: Left precentral gyrus | 229: Left postcentral gyrus | 5.1686 | 3.5244 | 0.0015 |
| 180: Left precentral gyrus | 249: Left supplementary motor area | 6.0460 | 3.7184 | 0.0013 |
| 180: Left precentral gyrus | 250: Left postcentral gyrus | 5.6775 | 3.8773 | 0.0011 |
| 182: Left hippocampus | 189: Left insula | 6.6624 | 3.4633 | 0.0017 |
| 195: Left inferior frontal gyrus, triangular part | 206: Left superior frontal gyrus, dorsolateral | 6.6703 | 3.9579 | 0.0011 |
| 195: Left inferior frontal gyrus, triangular part | 210: Left hippocampus | 9.3850 | 3.9082 | 0.0011 |
| 195: Left inferior frontal gyrus, triangular part | 222: Left insula | 6.4978 | 4.4642 | 0.0008 |
| 195: Left inferior frontal gyrus, triangular part | 229: Left postcentral gyrus | 8.9429 | 4.9984 | 0.0005 |
| 195: Left inferior frontal gyrus, triangular part | 241: Left superior temporal gyrus | 6.8523 | 3.8032 | 0.0012 |

Table S8 The functional connectivities associated with somatic symptoms in remission of MDD (continued)

| Functional connectivities | | Coefficient | t-value | *p*-value-FDR |
| --- | --- | --- | --- | --- |
| Region 1 | Region 2 |  |  |  |
| 204: Left middle frontal gyrus | 233: Left middle frontal gyrus | 4.4374 | 3.4976 | 0.0016 |
| 204: Left middle frontal gyrus | 234: Left thalamus | 6.9645 | 3.7149 | 0.0013 |
| 204: Left middle frontal gyrus | 250: Left postcentral gyrus | 4.8750 | 3.1569 | 0.0031 |
| 216: Left superior parietal gyrus | 244: Left anterior cingulate & paracingulate gyri | 7.7416 | 3.7938 | 0.0012 |
| 222: Left insula | 245: Left caudate nucleus | 5.5427 | 3.5203 | 0.0015 |
| 224: Left superior frontal gyrus, medial | 250: Left postcentral gyrus | 5.3783 | 3.5625 | 0.0014 |
| 227: Left middle frontal gyrus | 250: Left postcentral gyrus | 5.2371 | 3.3087 | 0.0022 |
| 241: Left superior temporal gyrus | 245: Left caudate nucleus | 6.9088 | 3.8468 | 0.0011 |

*p*-value-FDR: *p* value corrected by false discovery rate.

Table S9 The common functional connectivities of subclinical anxiety and somatic symptoms in remission of MDD

| Functional connectivities | | coefficient | | *p*-value-FDR |
| --- | --- | --- | --- | --- |
| Region 1 | Region 2 | Anxiety | Somatic |  |
| 1: Right posterior orbitofrontal gyrus | 173: Left temporal pole: superior temporal gyrus | 5.5407 | 8.9310 | < 0.01 |
| 1: Right posterior orbitofrontal gyrus | 247: Left middle occipital gyrus | 6.6991 | 8.6775 | < 0.01 |
| 7: Right gyrus rectus | 149: Left inferior parietal gyrus, excluding supramarginal and angular gyri | 6.6218 | 6.5947 | < 0.01 |
| 16: Right superior frontal gyrus, dorsolateral | 162: Left precentral gyrus | 5.5512 | 5.7440 | < 0.01 |
| 21: Right precentral gyrus | 180: Left precentral gyrus | 4.4243 | 5.1912 | < 0.01 |
| 38: Right temporal pole: middle temporal gyrus | 175: Left hippocampus | 4.8643 | 6.4120 | < 0.01 |
| 38: Right temporal pole: middle temporal gyrus | 222: Left insula | 5.6322 | 5.8176 | < 0.01 |
| 39: Right middle cingulate & paracingulate gyri | 222: Left insula | 6.6240 | 5.5039 | < 0.01 |
| 62: Right anterior cingulate & paracingulate gyri | 180: Left precentral gyrus | 5.4560 | 7.1969 | < 0.01 |
| 72: Right superior frontal gyrus, medial orbital (or ventromedial prefrontal cortex) | 149: Left inferior parietal gyrus, excluding supramarginal and angular gyri | 6.3681 | 6.7861 | < 0.01 |
| 84: Right insula | 173: Left temporal pole: superior temporal gyrus | 4.9448 | 6.1656 | < 0.01 |
| 84: Right insula | 212: Left fusiform gyrus | 5.4920 | 6.7448 | < 0.01 |
| 89: Right middle frontal gyrus | 227: Left middle frontal gyrus | 5.0809 | 5.3291 | < 0.01 |
| 102: Right superior frontal gyrus, dorsolateral | 222: Left insula | 5.9191 | 5.6036 | < 0.01 |
| 105: Right supplementary motor area | 162: Left precentral gyrus | 6.9882 | 7.2463 | < 0.01 |
| 105: Right supplementary motor area | 180: Left precentral gyrus | 5.7224 | 6.1926 | < 0.01 |
| 121: Right temporal pole: middle temporal gyrus | 222: Left insula | 5.2961 | 4.6492 | < 0.01 |
| 125: Right lenticular nucleus, putamen | 178: Left angular gyrus | 7.3679 | 8.4039 | < 0.01 |
| 128: Right cuneus | 184: Left inferior temporal gyrus | 5.5984 | 6.8899 | < 0.01 |

Table S9 The common functional connectivities of subclinical anxiety and somatic symptoms in remission of MDD (continued)

| Functional connectivities | | coefficient | | *p*-value-FDR |
| --- | --- | --- | --- | --- |
| Region 1 | Region 2 | Anxiety | Somatic |  |
| 129: Right parahippocampal gyrus | 168: Left superior temporal gyrus | 6.2710 | 6.8028 | < 0.01 |
| 129: Right parahippocampal gyrus | 187: Left lingual gyrus | 6.1159 | 7.6662 | < 0.01 |
| 138: Left precuneus | 151: Left insula | 8.7393 | 8.7990 | < 0.01 |
| 150: Left anterior cingulate & paracingulate gyri | 222: Left insula | 7.5426 | 7.6369 | < 0.01 |
| 195: Left inferior frontal gyrus, triangular part | 229: Left postcentral gyrus | 6.4367 | 8.9429 | < 0.01 |

*p*-value-FDR: *p* value corrected by false discovery rate.

Table S10 The common functional connectivities of subclinical depressive and somatic symptoms in remission of MDD

| Functional connectivities | | coefficient | | *p*-value-FDR |
| --- | --- | --- | --- | --- |
| Region 1 | Region 2 | Depression | Somatic |  |
| 16: Right superior frontal gyrus, dorsolateral | 250: Left postcentral gyrus | 8.6520 | 5.4383 | < 0.01 |
| 39: Right middle cingulate & paracingulate gyri | 188: Left precentral gyrus | 11.8880 | 6.7737 | < 0.01 |
| 44: Right postcentral gyrus | 164: Left middle frontal gyrus | 10.9564 | 7.7679 | < 0.01 |
| 62: Right anterior cingulate & paracingulate gyri | 125: Right lenticular nucleus, putamen | 8.5562 | 5.1596 | < 0.01 |
| 64: Right superior temporal gyrus | 173: Left temporal pole: superior temporal gyrus | 8.1612 | 4.9417 | < 0.01 |
| 77: Right middle frontal gyrus | 173: Left temporal pole: superior temporal gyrus | 12.5021 | 7.7072 | < 0.01 |
| 109: Right fusiform gyrus | 194: Left middle occipital gyrus | 10.2577 | 6.3040 | < 0.01 |
| 114: Right precentral gyrus | 220: Left temporal pole: superior temporal gyrus | 10.3734 | 6.4122 | < 0.01 |
| 125: Right lenticular nucleus, putamen | 158: Left precuneus | 9.8091 | 6.3616 | < 0.01 |
| 125: Right lenticular nucleus, putamen | 187: Left lingual gyrus | 9.7580 | 6.2629 | < 0.01 |
| 132: Right middle frontal gyrus | 178: Left angular gyrus | 10.3759 | 5.9661 | < 0.01 |
| 152: Left middle frontal gyrus | 250: Left postcentral gyrus | 9.6397 | 6.2214 | < 0.01 |
| 195: Left inferior frontal gyrus, triangular part | 229: Left postcentral gyrus | 10.7413 | 8.9429 | < 0.01 |

*p*-value-FDR: *p* value corrected by false discovery rate.

Table S11 The results of the chain mediation analysis of the effect of subclinical depressive symptoms on subclinical anxiety symptoms in healthy participants

|  | Effect | Boot SE | Boot LLCI | Boot ULCI |
| --- | --- | --- | --- | --- |
| Direct Effect | 0.3860 | 0.0446 | 0.3002 | 0.4771 |
| Total Indirect Effect | 0.0753 | 0.0268 | 0.0235 | 0.1290 |
| Ind1 | 0.0733 | 0.0263 | 0.0230 | 0.1260 |
| Ind2 | 0.0020 | 0.0011 | 0.0003 | 0.0043 |
| Ind1-Ind2 | 0.0714 | 0.0257 | 0.0225 | 0.1235 |

Boot SE: bootstrap standard error; Boot LLCI: bootstrap lower limit confidence interval; Boot ULCI: bootstrap upper limit confidence interval; Pair 55-234: the functional connectivity strength between the right medial superior frontal gyrus and the left thalamus; Ind1: Depression score → Somatic complaints score → Anxiety score; Ind2: Depression score → Pair 55-234 → Somatic complaints score →Anxiety score.

Table S12 The results of the chain mediation analysis of the effect of subclinical anxiety symptoms on subclinical depressive symptoms in healthy participants

|  | Effect | Boot SE | Boot LLCI | Boot ULCI |
| --- | --- | --- | --- | --- |
| Direct Effect | 0.5376 | 0.0573 | 0.4245 | 0.6485 |
| Total Indirect Effect | 0.2784 | 0.0450 | 0.1947 | 0.3712 |
| Ind1 | 0.2717 | 0.0446 | 0.1893 | 0.3641 |
| Ind2 | 0.0068 | 0.0042 | –0.0006 | 0.0156 |
| Ind1-Ind2 | 0.2649 | 0.0446 | 0.1827 | 0.3586 |

Boot SE: bootstrap standard error; Boot LLCI: bootstrap lower limit confidence interval; Boot ULCI: bootstrap upper limit confidence interval; Pair 55-234: the functional connectivity strength between the right medial superior frontal gyrus and the left thalamus; Ind1: Anxiety score → Somatic complaints score → Depression score; Ind2: Anxiety score → Somatic complaints score → Pair 55-234 → Depression score.

Table S13 The results of the chain mediation analysis of the effect of subclinical depressive symptoms on subclinical anxiety symptoms in remission of MDD

|  | Effect | Boot SE | Boot LLCI | Boot ULCI |
| --- | --- | --- | --- | --- |
| Direct Effect | 0.2539 | 0.0720 | 0.1025 | 0.3836 |
| Total Indirect Effect | 0.1860 | 0.0667 | 0.0704 | 0.3398 |
| Ind1 | –0.0098 | 0.0241 | –0.0471 | 0.0561 |
| Ind2 | –0.0223 | 0.0445 | –0.1234 | 0.0550 |
| Ind3 | 0.1553 | 0.0611 | 0.0467 | 0.2897 |
| Ind4 | 0.0191 | 0.0189 | –0.0113 | 0.0650 |
| Ind5 | 0.0437 | 0.0309 | 0.0024 | 0.1190 |
| Ind1-Ind2 | 0.0125 | 0.0360 | –0.0285 | 0.1143 |
| Ind1-Ind3 | –0.1650 | 0.0745 | –0.3122 | –0.0157 |
| Ind1-Ind4 | –0.0289 | 0.0305 | –0.0883 | 0.0376 |
| Ind1-Ind5 | –0.0534 | 0.0416 | –0.1323 | 0.0377 |
| Ind2-Ind3 | –0.1775 | 0.0937 | –0.3738 | –0.0047 |
| Ind2-Ind4 | –0.0414 | 0.0441 | –0.1388 | 0.0399 |
| Ind2-Ind5 | –0.0659 | 0.0635 | –0.2180 | 0.0303 |
| Ind3-Ind4 | 0.1361 | 0.0630 | 0.0297 | 0.2785 |
| Ind3-Ind5 | 0.1116 | 0.0643 | 0.0058 | 0.2542 |
| Ind4-Ind5 | –0.0245 | 0.0350 | –0.1140 | 0.0269 |

Boot SE: bootstrap standard error; Boot LLCI: bootstrap lower limit confidence interval; Boot ULCI: bootstrap upper limit confidence interval; AS-FC: the mean strength of common functional connectivities of subclinical anxiety and somatic symptoms after removing the functional connectivity between the triangular part of the left inferior frontal gyrus and the left postcentral gyrus; DS-FC: the mean strength of common functional connectivities of subclinical depressive and somatic symptoms after removing the functional connectivity between the triangular part of the left inferior frontal gyrus and the left postcentral gyrus; Ind1: Depression score → Somatic complaints score → Anxiety score; Ind2: Depression score → DS-FC → Somatic complaints score → Anxiety score; Ind3: Depression score → DS-FC → AS-FC → Anxiety score; Ind4: Depression score → Somatic complaints score → AS-FC →Anxiety score; Ind5: Depression score→ DS-FC → Somatic complaints score → AS-FC → Anxiety score.

Table S14 The results of the chain mediation analysis of the effect of subclinical anxiety symptoms on subclinical depressive symptoms in remission of MDD

|  | Effect | Boot SE | Boot LLCI | Boot ULCI |
| --- | --- | --- | --- | --- |
| Direct Effect | 0.5859 | 0.2341 | 0.1023 | 1.0348 |
| Total Indirect Effect | 0.5465 | 0.2084 | 0.2160 | 1.0364 |
| Ind1 | 0.0067 | 0.0539 | –0.0551 | 0.1597 |
| Ind2 | 0.0384 | 0.1103 | –0.1681 | 0.2712 |
| Ind3 | 0.4186 | 0.1545 | 0.1355 | 0.7488 |
| Ind4 | 0.0124 | 0.0357 | –0.0521 | 0.0896 |
| Ind5 | 0.0704 | 0.0788 | –0.0411 | 0.2723 |
| Ind1-Ind2 | –0.0317 | 0.1011 | –0.2426 | 0.1699 |
| Ind1-Ind3 | –0.4118 | 0.1662 | –0.7419 | –0.0810 |
| Ind1-Ind4 | –0.0056 | 0.0529 | –0.0850 | 0.1333 |
| Ind1-Ind5 | –0.0637 | 0.0981 | –0.2775 | 0.1264 |
| Ind2-Ind3 | –0.3802 | 0.2089 | –0.7858 | 0.0356 |
| Ind2-Ind4 | 0.0260 | 0.1100 | –0.1749 | 0.2674 |
| Ind2-Ind5 | –0.0320 | 0.1394 | –0.3431 | 0.2055 |
| Ind3-Ind4 | 0.4062 | 0.1611 | 0.1117 | 0.7558 |
| Ind3-Ind5 | 0.3482 | 0.1731 | 0.0436 | 0.7181 |
| Ind4-Ind5 | –0.0580 | 0.0859 | –0.2873 | 0.0364 |

Boot SE: bootstrap standard error; Boot LLCI: bootstrap lower limit confidence interval; Boot ULCI: bootstrap upper limit confidence interval; AS-FC: the mean strength of common functional connectivities of subclinical anxiety and somatic symptoms after removing the functional connectivity between the triangular part of the left inferior frontal gyrus and the left postcentral gyrus; DS-FC: the mean strength of common functional connectivities of subclinical depressive and somatic symptoms after removing the functional connectivity between the triangular part of the left inferior frontal gyrus and the left postcentral gyrus; Ind1: Anxiety score → Somatic complaints score → Depression score; Ind2: Anxiety score → AS-FC → Somatic complaints score → Depression score; Ind3: Anxiety score → AS-FC → DS-FC → Depression score; Ind4: Anxiety score → Somatic complaints score → DS-FC → Depression score; Ind5: Anxiety score → AS-FC → Somatic complaints score → DS-FC → Depression score.

Fig. S1 The flow chart of identifying healthy participants from the HCP

HCP-Young Adult sample size: 1206

Participants without MDD history: 1060

Participants with DSM-Ⅳ Anxiety Problems subscale ≥ 1: 966

Participants with DSM-Ⅳ Depressive Problems subscale ≥ 1: 906

Participants with four runs of rs-fMRI: 752

Healthy participants meeting the purpose of this study: 466

Participants with missing MDD history data: 33

Participants with MDD history: 113

Participants with missing DSM-Ⅳ Anxiety Problems subscale data: 8

Participants with DSM-Ⅳ Anxiety Problems subscale = 0: 86

Participants with DSM-Ⅳ Depressive Problems subscale = 0: 60

Participants with uncompleted rfMRI: 154

Participants with marijuana dependence: 69

Participants with alcohol abuse/dependence: 99

Participants with positive breathalyzer/drug test results: 59

Participants with history of panic disorder/agoraphobia: 52

Participants with missing race data: 5

Participants with missing data of parental history of neuropsychiatric disorders: 2

Legend: HCP: Human Connectome Project; MDD: Major depressive disorder; rs-fMRI: Resting-state functional magnetic resonance imaging.

Fig. S2 The flow chart of identifying participants in remission of MDD from the HCP

HCP-Young Adult sample size: 1206

Participants with MDD history: 113

Participants with DSM-Ⅳ Anxiety Problems subscale ≥ 1: 112

Participants with DSM-Ⅳ Depressive Problems subscale ≥ 1: 110

Participants with four runs of rs-fMRI: 87

Participants in remission of MDD meeting the purpose of this study: 53

Participants with missing MDD history data: 33

Participants without MDD history: 1060

Participants with DSM-Ⅳ Anxiety Problems subscale = 0: 1

Participants with DSM-Ⅳ Depressive Problems subscale = 0: 2

Participants with uncompleted rfMRI: 23

Participants with marijuana dependence: 13

Participants with alcohol abuse/dependence: 18

Participants with positive breathalyzer/drug test results: 2

Participants with missing race data: 1

Legend: HCP: Human Connectome Project; MDD: Major depressive disorder; rs-fMRI: Resting-state functional magnetic resonance imaging.

Fig. S3 The chain mediation models of the effect of subclinical anxiety symptoms on subclinical depressive symptoms

Somatic complaints score

Anxiety score

Depression score

Pair 55-234

a.

β = –0.6950

β = 0.4991**

β = 0.5376**

β = –0.0179**

β = 0.5444**

Somatic complaints score

Anxiety score

Depression score

AS-FC

DS-FC

b.

β = 13.0657**

β = 0.0715

β = 0.5859**

β = 0.0100

β = 0.6895**

β = 11.5695**

β = 0.0944

β = 0.0465**

Legend: ***p* < 0.01; a: The chain mediation model of the effect of subclinical anxiety symptoms on subclinical depressive symptoms in healthy participants; b: The chain mediation model of the effect of subclinical anxiety symptoms on subclinical depressive symptoms in remission of major depressive disorder; β: regression coefficient; Pair 55-234: the strength of the functional connectivity between the right medial superior frontal gyrus and the left thalamus; AS-FC: the mean strength of common functional connectivities of subclinical anxiety and somatic symptoms after removing the functional connectivity between the triangular part of the left inferior frontal gyrus and the left postcentral gyrus; DS-FC: the mean strength of common functional connectivities of subclinical depressive and somatic symptoms after removing the functional connectivity between the triangular part of the left inferior frontal gyrus and the left postcentral gyrus.
